# Unveiling the Critical Pathways of Hydroxyl Radical Formation in Breakpoint Chlorination: The Role of Trichloramine and Dichloramine Interactions

**DOI:** 10.1021/acs.est.4c08403

**Published:** 2024-11-11

**Authors:** Yi-Hsueh Chuang, Chia-Shun Chou, Yi-Lin Chu

**Affiliations:** Institute of Environmental Engineering, National Yang Ming Chiao Tung University, Hsinchu city 30010, Taiwan

**Keywords:** breakpoint chlorination, trichloramine, dichloramine, hydroxyl radical

## Abstract

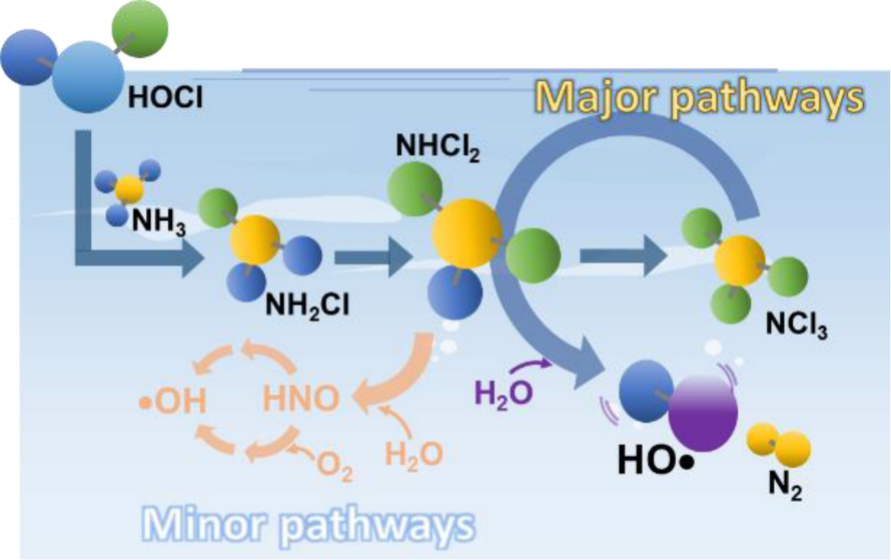

Chlorination of ammonia or chloramine-containing waters induces breakpoint chlorination reactions, producing a hydroxyl radical (•OH), but enhances the formation of undesirable *N*-nitrosamines. The prevailing view attributes •OH formation to a nitrosyl intermediate derived from the hydrolysis of dichloramine, but this pathway is unlikely at neutral or acidic pH. This study reveals a novel mechanism where •OH is generated via interactions between trichloramine (NCl_3_) and dichloramine (NHCl_2_), which also form nitrosation agents. Our experiments demonstrated that the NCl_3_–NHCl_2_ interaction degrades micropollutants with kinetics 2–3 times faster than breakpoint chlorination. Using electron paramagnetic resonance, we detected •OH in the NCl_3_–NHCl_2_ reaction. Micropollutant removal was unimpaired under low dissolved oxygen (O_2(aq)_) conditions, aligning with negligible O_2(aq)_ changes during the NCl_3_–NHCl_2_ reaction and suggesting O_2(aq)_ does not participate in •OH formation. Using benzene as a probe in ^18^O-labeled H_2_O, we confirmed water contributes to the oxygen source of •OH in NCl_3_–NHCl_2_ interactions, through which parallel reactions occur, leading to the formation of one mole of •OH alongside 1.92 mol of N_2_. A kinetic model developed in this study accurately predicted •OH and N_2_ and demonstrated the NCl_3_–NHCl_2_ interaction as the primary pathway for •OH formation in breakpoint chlorination, providing new insights into breakpoint chemistry.

## Abstract 2

This study reveals that the reaction between trichloramine and dichloramine accounts for nearly all •OH formation in breakpoint chlorination, challenging the prevailing view that attributes •OH generation primarily to dichloramine hydrolysis.

## Introduction

Ammonia and inorganic chloramines are frequently present in potable reuse systems and drinking water at concentrations ranging from sub-mg/L to mg/L (as N or Cl_2_).^1−3^ Chlorine, typically added in the form of sodium hypochlorite or calcium hypochlorite, is used to reduce the acute health risks associated with waterborne pathogens. The chlorination of water containing ammonia or chloramines induces “breakpoint chlorination” reactions when the chlorine-to-nitrogen molar ratio exceeds 1.5 (Cl_2_/N > 1.5). These reactions include hypochlorous acid transforming ammonia into inorganic chloramines and the decomposition of inorganic chloramines, resulting in a net loss of total chlorine.

Although breakpoint chlorination has long been used to eliminate ammonia nitrogen and maintain free chlorine residuals in water treatment systems,^4,5^ recent studies suggest it may generate hydroxyl radicals (•OH) and reactive nitrogen species, including peroxynitrous acid/peroxynitrite (HOONO/OONO^–^), nitric oxide (•NO), and nitrogen dioxide (•NO_2_). These reactive species, especially •OH, aid chemical^6−8^ or biological^9^ micropollutant removals, potentially lowering the burden of the water treatment trains. However, breakpoint chlorination also inevitably enhances the formation of harmful nitrosamines,^10^ prompting the need for caution in balancing disinfection efficiency with byproduct formation. A deeper understanding of the chemistry behind the formation of reactive species and nitrosamines is critical to achieving this balance.

Separate studies of the degradation of HOCl-resistant compounds show that micropollutant degradation patterns mirror those of nitrosamine formation. For instance, the degradation rates of atrazine,^6^ benzotriazole,^6^ and 1,4-dioxane^7^ follow a volcano-shaped trend at Cl_2_/N ratios from 0 to 4 at neutral pH. Removals increase as the Cl_2_/N ratio rises to about 1.5–2 and decrease thereafter. This trend aligns with nitrosamine formation observed during chlorination of ammonia-containing water with dimethylamine (DMA) precursor^10^ (see additional discussion in Text S1 in the Supporting Information). Additionally, micropollutant removals correlate with total chlorine loss in breakpoint chlorination, matching nitrosamine formation patterns^11^ (Text S1). These findings suggest that some breakpoint chlorination reactions proceed via parallel pathways, forming reactive species and nitrosating agents while simultaneously resulting in a loss of total chlorine.

The widely used unified kinetics model (UF; Table S1) has been demonstrated to accurately predict the changes in concentrations of HOCl/OCl^–^ and inorganic chloramines at Cl_2_/N ranging from 0.2 to 2. However, none of the reactions in the UF model suggest the formation of •OH or transient nitrogenous species. Until now, the prevailing view^6−8,10,12,13^ links •OH and transient nitrogen species generation in breakpoint chlorination to nitroxyl (HNO) formation following NHCl_2_ hydrolysis (Reactions R1–R5). HNO was postulated to be an intermediate from the hydrolysis of NHCl_2_ during breakpoint chlorination (R1).^14^ At neutral pH, nitroxyl anion (NO^–^; p*K*_a_ of HNO = ∼7)^15^ reacts with dissolved oxygen (O_2(aq)_) to form peroxynitrite (R2), which later decomposes to •NO and •O_2_^–^ (R5).^16^ Peroxynitrous acid (HOONO, p*K*_a_ = 6.8) can either isomerize to nitric acid (HNO_3_; R3) or decay to •OH and •NO_2_ (∼28%; R4).^16^ These reaction schemes underscore the role of O_2(aq)_ in forming •OH and transient •NO_2_/•NO species, concurring with previous research showing O_2_ consumption during the reaction of NH_4_^+^ with HOCl.^13^

Alternatively, •OH can form via dimerization of two HNO molecules to *cis*-hyponitrous acid (HO—N=N—OH) (R6) followed by a concomitant azo-type homolytic fission, leading to a N_2_ and two •OH (R7).^17,18^ This O_2_-independent pathway for •OH formation has been well studied in biochemistry, with speculation that *in vivo* generated HNO/NO^–^ may damage DNA.^19^ A recent study utilized terephthalate as a probe compound to trace the oxygen source of the •OH and demonstrated that over 90% of the oxygen in •OH comes from H_2_O rather than dissolved O_2_ during breakpoint chlorination.^8^ This suggests that the R6–R7 pathway predominates in •OH formation.
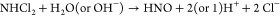
R1
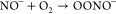
R2
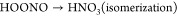
R3
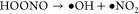
R4
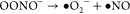
R5
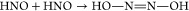
R6
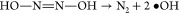
R7

Both the O_2_-dependent (R1–R5) and the O_2_-independent (R6–R7) pathways stem from NHCl_2_ hydrolysis (R1). NHCl_2_ hydrolytic decay, however, is slow at neutral pH, with a half-life of ∼17 h at pH 7.0 (*k* = 1.1 × 10^–5^ s^–1^ at pH 7.0).^20^ Using the UF model, our previous study showed that the cumulative products from NHCl_2_ hydrolysis are at 10^–8^ M levels when treating 100 μM HOCl with 50 μM NH_4_^+^ at pH 7 for 20 min.^11^ This concentration is about two orders of magnitude lower than the micropollutant (nitrobenzene and benzoate) concentrations decomposed during similar breakpoint chlorination reactions (i.e., 2 μM micropollutant removal with 70 μM NH_4_^+^ and 177 μM HOCl at pH 7 for 20 min).^12^ Given that nitrobenzene and benzoate are resistant to reactive nitrogen species,^8^ these findings question the significance of HNO-derived HOONO/OONO^–^ (R1–R5) or *cis*-hyponitrous acid (R6–R7) in •OH formation during breakpoint reactions.

**Scheme 1:**
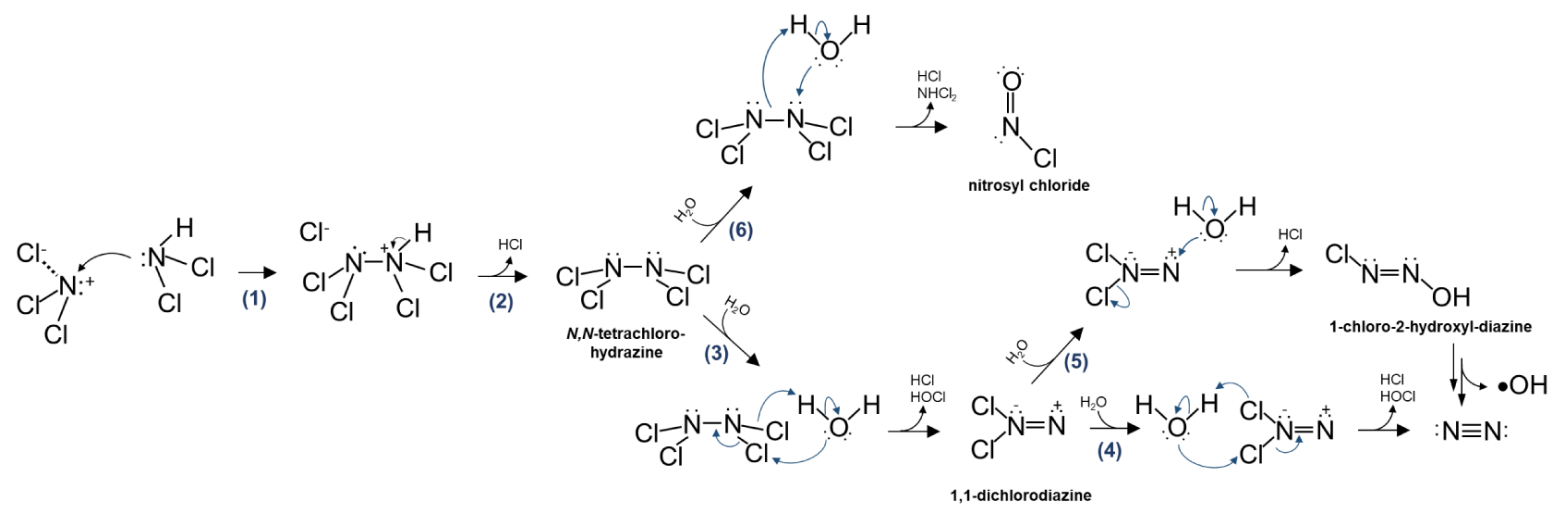
Proposed Reaction Pathways for the Reaction of NCl_3_ with NHCl_2_ That Result in ClNO, •OH, and N_2_ End Products, and Reactions R1–R4 and Reaction R6 Were Adapted from the Literature^11,22^

Our previous study showed that enhanced nitrosamine formation at Cl_2_/N ratios above the breakpoint is due to the reaction of NCl_3_ with NHCl_2_, through which nitrosyl chloride (ClNO) forms as a nitrosating agent. Given that nitrosamine formation patterns mirror those observed in micropollutant removal, we hypothesize that the interaction between NCl_3_ and NHCl_2_ may also result in the generation of •OH. A couple of studies have evaluated the pathways and products of the NCl_3_ reaction with NHCl_2_,^11,21,22^ where the mechanism involves the nucleophilic attack of the nitrogen atom in NHCl_2_ on NCl_3_, producing an *N*,*N*-tetrachlorohydrazine (Cl_2_N–NCl_2_) intermediate (Reactions R1 and R2 in Scheme 1). Quantum chemical calculations using density functional theory suggest that chlorine transfer to H_2_O yields HOCl and 1,1-dichlorodiazine (Cl_2_N=N), which subsequently hydrolyzes to HOCl, HCl, and N_2_ (Reactions R3 and R4 in Scheme 1). Alternatively, the hydrolysis of the Cl_2_N–NCl_2_ intermediate can yield ClNO, a potent nitrosating agent, through nucleophilic attack by H_2_O or OH^–^ followed by dehydrochlorination (Reaction R6 in Scheme 1). Despite the insights provided by existing literature into these reaction pathways, the proposed mechanisms do not indicate the formation of •OH. Additionally, previous research using electron paramagnetic resonance (EPR) with the 5,5-dimethyl-1-pyrroline *N*-oxide (DMPO) spin trap has not detected •OH during breakpoint chlorination. Therefore, the potential generation of •OH during the NCl_3_–NHCl_2_ interaction and its role in micropollutant removal remain unclear, necessitating further investigation.

The objectives of this study are to (1) investigate the significance of •OH generated in breakpoint chlorination in degrading micropollutants and (2) explore the mechanism of •OH formation in breakpoint chlorination. While our results demonstrated that parallel reactions occur during the reaction of NCl_3_ with NHCl_2_ which produces •OH and N_2_, evaluating the contribution of the NCl_3_–NHCl_2_ interaction to •OH formation during breakpoint chlorination necessitates the development of a kinetic model. Therefore, the third objective is to combine the experimental data with an updated kinetic model to understand the contribution of the NCl_3_–NHCl_2_ interaction on •OH formation during breakpoint chlorination.

## Materials and Methods

### Chemicals and Reagents

All chemicals used in this study are listed in Table S2.

### Chloramine Stock Solutions

NH_2_Cl and NHCl_2_ were prepared freshly. The NH_2_Cl stock solution was prepared by adding a 40 mM NaOCl dropwise to a 48 mM NH_4_Cl solution and stirred continuously at a 1:1.2 Cl_2_:N molar ratio without buffering, resulting in a final pH of ∼9.5 for the solution.^23^ Stock solutions of NHCl_2_ were prepared by adjusting the pH of a 3.5 mM NH_2_Cl solution to 3.8 with 0.2 M phosphoric acid and maintaining this condition for 30 min. The solution was then stored in an ice bath for an additional hour to allow the NH_2_Cl disproportionation reaction (2 NH_2_Cl + H^+^ → NHCl_2_ + NH_4_^+^) to complete, leaving a solution containing NHCl_2_ and NH_4_^+^ at approximately a 1:1.4 molar ratio. The NCl_3_ stock solution was prepared by mixing a 31 mM HOCl solution (pH 7) with a 10 mM NH_4_^+^ solution (pH 4; headspace-free) and allowing the mixture to age for 18–24 h.^24^ A Cary 60 UV–visible spectrophotometer was used to standardize the three inorganic chloramine stock solutions.^24,25^ Since HOCl inevitably coexists in NCl_3_ solutions at molar ratios of approximately 0.4–0.6:1 HOCl:NCl_3_ (measured by the DPD (*N*,*N*-diethyl-p-phenylenediamine oxalate) colorimetric method),^26,27^ a quantitative amount of H_2_O_2_ (∼1.05 molar ratio) was added to quench the residual HOCl before experiments. The peroxynitrite stock solution was prepared following the procedure described in the literature.^28^

### Degradation of Model Compounds during Breakpoint Chlorination or the Reaction of NCl_3_ with NHCl_2_

Five model micropollutants (1,4-dioxane, *N*,*N*-diethyl-meta-toluamide (DEET), benzoate, nitrobenzene, and caffeine) were selected to evaluate their degradation during breakpoint chlorination and reactions with NCl_3_ and NHCl_2_. These compounds exhibit distinct reactivities toward reactive species in breakpoint chlorination. Table S3 summarizes the rate constants for the radicals. The *k*_•OH_ values range from 2.5 × 10^9^ to 7.5 × 10^9^ M^–1^ s^–1^. While 1,4-dioxane is not highly reactive with •Cl (*k*_•Cl_ = 4.4 × 10^6^ M^–1^ s^–1^), the *k*_•Cl_ values span from 5.2 × 10^8^ M^–1^ s^–1^ for nitrobenzene to 3.9 × 10^10^ M^–1^ s^–1^ for caffeine. Caffeine also reacts with •ClO radicals, with a *k*_•ClO_ of 1.03 × 10^8^ M^–1^ s^–1^.^29^ DEET reacts with reactive nitrogen species (RNS) such as HOONO/OONO^–^, •NO, and •NO_2_^–^, with a collective rate constant of ∼1 × 10^9^ M^–1^ s^–1^.^8^

The experimental details for breakpoint chlorination and the reaction of NCl_3_ with NHCl_2_ are available in our previous study.^11^ Briefly, breakpoint chlorination experiments were conducted using NaOCl and NH_4_^+^ at target Cl_2_/N molar ratios with micropollutants at sub-μM levels (0.2–0.5 μM). To initiate the reaction of NCl_3_ with NHCl_2_, an aliquot of NCl_3_ stock solution was added to a phosphate buffer containing five compounds, each at concentrations of 0.2 or 0.4 μM, in a 60 mL vial at the desired concentration, followed by the addition of H_2_O_2_. The solution was gently mixed by pipetting after adding NHCl_2_, while minimizing the headspace to prevent NCl_3_ loss through volatilization. Note that the addition of H_2_O_2_ effectively neutralizes HOCl without compromising the stability of NCl_3_ or NHCl_2_, as confirmed spectrometrically.^11^ Additionally, although a slight excess of H_2_O_2_ was present, it did not impact •OH formation during NCl_3_–NHCl_2_ interactions (Figure S8). A slight excess of ascorbic acid or sodium thiosulfate (1.1:1 molar ratio) was used to quench the residual chlorine/chloramines after the reaction. The 20 mM phosphate buffer maintained a stable pH throughout the experiments (<0.2 unit).

In the experiments involving the reaction between NCl_3_ and NHCl_2_, the molar ratio of NCl_3_ to NHCl_2_ was ≤5:2 in most cases, despite variations in their concentrations. This ratio was determined based on the observation that one mole of NHCl_2_ typically consumes approximately 2.6 mol of NCl_3_,^11^ driven by the partial reformation of NHCl_2_ during the NCl_3_–NHCl_2_ interactions. Maintaining a molar ratio of NCl_3_ to NHCl_2_ ≤ 5:2 ensures the complete degradation of NCl_3_ postreaction. Experiments examining nitrosamine formation^11^ and micropollutant removals (as discussed below) produced similar results when the NCl_3_ to NHCl_2_ molar ratio was ≤5:2.

### Tracing •OH Oxygen Origin during NCl_3_–NHCl_2_ Interactions

Benzene was used as a probe to trace the origin of •OH oxygen during NCl_3_–NHCl_2_ interactions. To initiate the experiments, 100 μM benzene solution prepared in ^18^O–H_2_O (1 mL, 97% ^18^O atom; Aladdin Scientific, Shanghai, China) was buffered with 20 mM phosphates at pH 7. This solution was treated with 50 μM NCl_3_ and 30 μM NHCl_2_ for 3 min, followed by the addition of 200 μM thiosulfate to quench the reaction. Due to the introduction of approximately 170 μL of nonisotope-labeled H_2_O from the benzene, NCl_3_, and NHCl_2_ solutions (all prepared in deionized water), the final reaction mixture consisted of approximately 83% ^18^O-labeled H_2_O.

### Nitrogen Balance Experiments during the Reaction of NCl_3_ with NHCl_2_

Gaseous and ionic nitrogen products were quantified in the reaction of NCl_3_ with NHCl_2_. Experiments were conducted using four samples, each containing a solution of ^15^N-NCl_3_ at the desired concentration, along with a stoichiometric amount of H_2_O_2_ to quench the residual HOCl in the NCl_3_. The pH was stabilized using a 20 mM phosphate buffer. The sample vials were immediately sealed following the addition of ^15^N-NHCl_2_ at the target concentration (which contained background ammonia at a molar concentration 1.4 times of the NHCl_2_). After 20 min of reaction time, at which ^15^N-NCl_3_ was completely consumed, two of the four samples were sacrificed for analyses of chloramine concentrations using the DPD method. The remaining two samples were subjected to analyses of N_2_, N_2_O, NO_2_^–^, NO_3_^–^, and ammonia following the addition of the thiosulfate quencher, as detailed in Text S2. Note that ammonia measured in the thiosulfate-quenched samples encompassed NH_2_Cl and NHCl_2_ reduced by the thiosulfate quencher.

For the analyses of N_2_ and N_2_O, the sample vial was kept at room temperature (25 ± 1 °C), vigorously shaken for 1 min, and allowed to stand for 30 min prior to sampling; these approaches facilitate the equilibrium of volatile nitrogen products (N_2_, N_2_O) between gaseous phase and water phase. An aliquot (5 μL) of the headspace sample was taken using a microsyringe and was injected into an Agilent 7890N GC equipped with an RTX-200 column and an Agilent 5977A mass spectrometer. Analytes were quantified under electron impact ionization in single ion monitoring mode (*m*/*z* of 28 for ^14^N–N_2_, *m*/*z* 30 for ^15^N–N_2_, and *m*/*z* 46 for ^15^N–N_2_O). The ratio of ^15^N–N_2_/^14^N–N_2_ in the gaseous sample was calculated based on the area counts for *m*/*z* 30 and *m*/*z* 28. The partial pressure of ^15^N–N_2_ can be calculated by multiplying the partial pressure of ^14^N–N_2_ (0.78 atm) by the ratio of ^15^N–N_2_/^14^N–N_2_. The analytical method was validated using a series of standard samples prepared by ^15^N–N_2_ (Sigma-Aldrich, 98%) with ambient air (Text S2). A Henry’s constant of 6.4 × 10^–6^ mol/m^3^/Pa for N_2_ at 25 °C was used to calculate the concentration of dissolved N_2_ in the solution.^30^ A similar approach was applied for the quantification of N_2_O, as specified in Text S2.

### Analytical Method

Nitrite, nitrate, and ammonia were analyzed by using a Dionex Aquion ion chromatograph with a conductivity detector. A Bruker Elexsys E580 EPR spectrometer was used to analyze the transient reactive species in the reaction between NCl_3_ and NHCl_2_. DMPO was used as a spin-trapping agent for •OH.^31^ Benzene concentration was analyzed using an Agilent GC (7890N)-MS (5977A) instrument (detailed in Text S3). Phenol formed from the reaction of benzene and •OH was measured using an Agilent HPLC (1260 II) coupled with a UV detector. While *tert*-butanol was used as an additional probe for tracking •OH formation, forming formaldehyde as the primary product, formaldehyde was quantified using an HPLC method following derivatization.^32^

### Kinetic Modeling

A kinetic model derived from the revised UF model (Table S6; detailed in later sections) was implemented using Kintecus 6.8.^33^ This model was checked by a computer program DETBAL,^34^ and no illegal loop was found.

## Results and Discussion

### Total Chlorine Loss, Nitrosamine Formation, and Micropollutant Removals in Breakpoint Chlorination

Initial experiments conducted across a pH range of 5.8–8.3 revealed breakpoints at Cl_2_/N molar ratios of approximately 1.8–2 (Figure S4). At a Cl_2_/N molar ratio of 2, total chlorine concentrations dropped considerably within the first 20 min across pH 5.8–8.3, and the patterns of total chlorine concentration coincided with micropollutant removals (Figure S5). Moreover, with the exception of caffeine, micropollutant removals at 20 min generally peaked at a Cl_2_/N of 1.8–2 (Figure S6), concurring with prior findings.^6,7^ Removals were generally higher at pH 7 and 5.8 (30–50%) than at pH 8.3, where negligible removals (6–20%) were observed except for caffeine. Caffeine removals were ∼100% at pH 8.3, compared to 55% at pH 5.8 (Figure S6).

**Figure 1:**
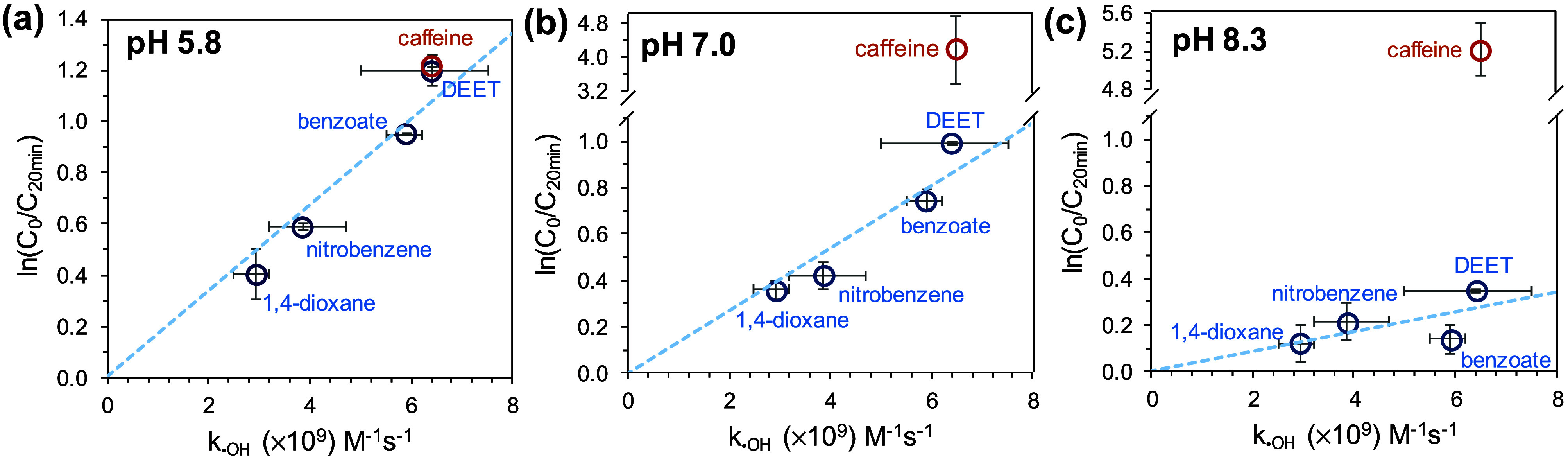
Relationship between *k*_•OH_ for compounds and ln[C]_0_/[C]_20 min_ for compounds in the treatments of the mixture of five micropollutants by breakpoint chlorination at (a) pH 5.8, (b) pH 7.0, and (c) pH 8.3. [HOCl]_0_ = 100 μM, [NH_4_^+^]_0_ = 50 μM. The pH of the solutions was maintained by 10 mM phosphates. [1,4-dioxane]_0_ = [DEET]_0_ = 0.2 μM, [benzoate]_0_ = [nitrobenzene]_0_ = [caffeine]_0_ = 0.4 μM. Dashed lines represent the linear regression for 1,4-dioxane, nitrobenzene, benzoate, and DEET (*R*^2^ = 0.92 for pH 5.8, 0.88 for pH 7.0, and 0.34 for pH 8.3). The *x*-axis error bar represents the range of *k*_•OH_ reported in the literature, while the *y*-axis error bars denote the range of experimental data (*n* = 2).

During the treatment of the mixture of micropollutants by breakpoint chlorination, the temporal change in concentration for a given compound (−d[C]/d*t*) can be mathematically expressed as shown in eq 1. Here, [C] represents the compound’s concentration, while *k*_C,•OH_ and *k*_C,•R_ are the rate constants for reactions with •OH and other reactive species (•R), respectively. Integrating eq 1 yields eq 2, which suggests a linear relationship between −ln(C_0_/C) and *k*_C,•OH_ if the contributions of other reactive species are negligible (i.e., minimal  in eq 2). Indeed, plotting ln([C]_0_/[C]_20 min_) for micropollutants against the *k*_C,•OH_ for micropollutants shows a linear relationship for all but caffeine at pH 5.8 or 7 (Figure 1), suggesting that •OH is the main reactive species degrading 1,4-dioxane, DEET, benzoate, and nitrobenzene in breakpoint chlorination. A more scattered relationship (*R*^2^ = 0.34) at pH 8.3 is likely due to negligible removals from radical scavenging by OCl^–^, which predominates at pH 8.3 (p*K*_a_ for HOCl = 7.4) and has a *k*_•OH_ value 5-fold higher than HOCl.^35,36^ The removal of caffeine significantly deviated from the other four compounds, especially at alkaline pH (Figure 1c). This suggests that radicals generated from •OH scavenging by OCl^–^ (e.g., •ClO; Reaction R8) became pronounced in caffeine’s degradation, as multiple studies have confirmed the importance of •ClO on caffeine degradation.^29,37,38^
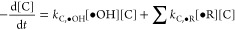
1
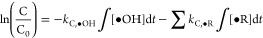
2
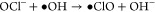
R8

To investigate intricate relationships between •OH generation, nitrosating agent formation, and chlorine loss during breakpoint chlorination, we used a solution containing 10 μM *N*-chlorodimethylamine (Cl-DMA) as a model precursor,^11,39^ along with 0.4 μM each of 1,4-dioxane and DEET. 1,4-Dioxane and DEET were chosen to represent compounds with *k*_•OH_ at the lower (2.9 × 10^9^ M^–1^ s^–1^) and upper (6.4 × 10^9^ M^–1^ s^–1^) ends of the range tested in this study. The solution was treated with 150 μM HOCl and 75 μM NH_4_^+^ at pH 5.8, 7, and 8.3. Results revealed significant interdependencies among total Cl_2_ losses (Δ[Cl_2_]_tot_), NDMA formation (Δ[NDMA]), and degradation of DEET and 1,4-dioxane (Δ[DEET] or Δ[1,4-dioxane]) over 20 min (Figure S7). NDMA formation normalized to Cl_2_ loss (i.e., the slopes of Δ[NDMA]/Δ[Cl_2_]_tot_) was about one-third lower at pH 5.8 compared to pH 7, while micropollutant removals at pH 5.8 were roughly double those at pH 7. This suggests that •OH and nitrosating agents are generated from intricate cross-reactions associated with chlorine loss, with acidic pH favoring •OH formation over that of nitrosating agents. Both micropollutant removals and NDMA formation decreased as pH increased from 7 to 8.3, likely due to OCl^–^ scavenging reactions. Although NDMA may degrade via reactions with •OH, its impact is negligible given NDMA’s low concentration (<30 nM) and lower *k*_•OH_ (3.3 × 10^8^ M^–1^ s^–1^) compared to micropollutants.

### Micropollutant Removals during the Reaction of NCl_3_ with NHCl_2_

Experiments assessed the potential of NCl_3_–NHCl_2_ interactions to generate radicals or reactive species for micropollutant degradation. Results showed more rapid degradation when micropollutants were treated with 50 μM NCl_3_ and 50 μM NHCl_2_ (equivalent to 250 μM HOCl and 100 μM NH_4_^+^), compared to treatment by breakpoint chlorination with the same chlorine equivalent at a Cl_2_/N ratio of 2 (Figure S8). For example, DEET exhibited a log removal of 0.88 ± 0.01 within 3 min at pH 7, significantly higher than the log removal of 0.41 ± 0.01 after 10 min with 250 μM HOCl and 125 μM NH_4_^+^ at the same pH. The degradation patterns of micropollutants during NCl_3_–NHCl_2_ treatment aligned with NCl_3_ decomposition (Figure S8), supporting the generation of reactive species in the reaction of NCl_3_ with NHCl_2_.

Previous research has reported the high reactivity of NCl_3_ with certain compounds, with reported *k* reaching approximately 10^3^ M^–1^ s^–1^ for aromatic compounds.^40^ To assess to what extent NCl_3_ degrades compounds in the NCl_3_–NHCl_2_ experiments, we experimentally measured the reactivities of NCl_3_ for the five compounds investigated (details are provided in Text S4). While 1,4-dioxane and DEET exhibited low reactivity with NCl_3_, nitrobenzene, benzoate, and caffeine featured  values of 2.1 ± 0.5, 1.9 ± 0.3, and 5.4 ± 0.6 M^–1^ s^–1^, respectively. However, degradation of these compounds due to reactions with NCl_3_ was negligible (<0.5%) during the NCl_3_–NHCl_2_ treatments, primarily due to the rapid decomposition of NCl_3_ when reacting with NHCl_2_ (detailed in Text S4). The results also demonstrated a low reactivity between NHCl_2_ and these compounds (Figure S9b).

**Figure 2:**
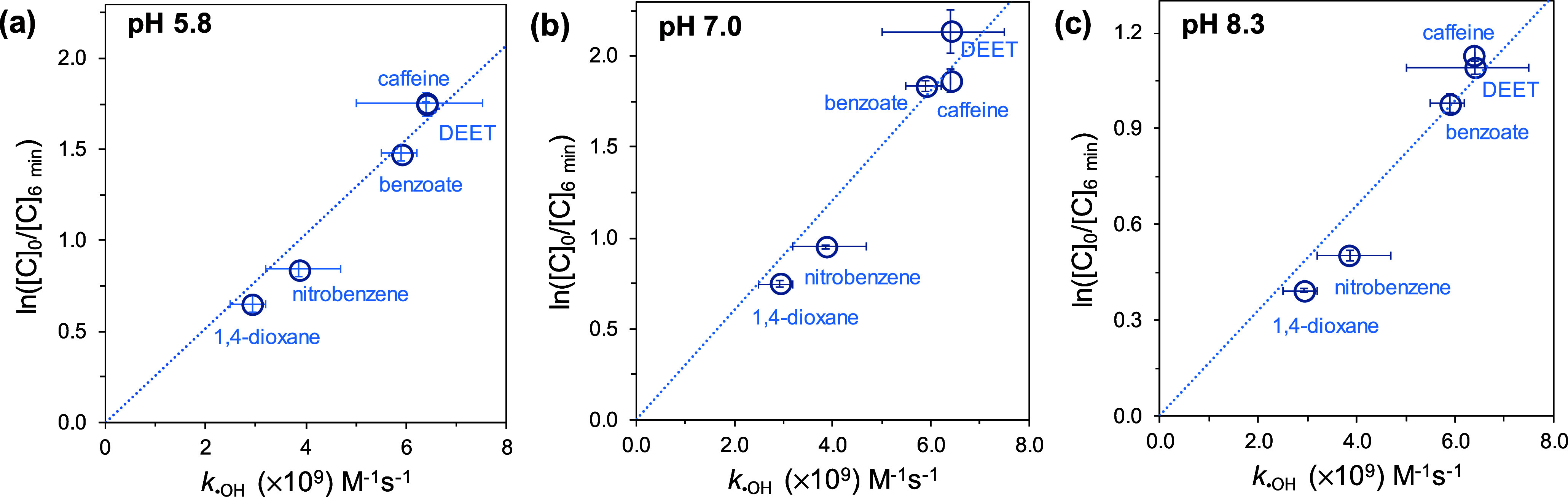
Relationship between *k*_•OH_ for compounds and ln([C]_0_/[C]_6 min_) for compounds in the treatments of the mixture of five micropollutants by 50 μM NCl_3_ + 50 μM NHCl_2_ at (a) pH 5.8, (b) pH 7.0, and (c) pH 8.3. The pH of the solutions was maintained by using 20 mM phosphates. [1,4-dioxane]_0_ = [DEET]_0_ = 0.2 μM, [benzoate]_0_ = [nitrobenzene]_0_ = [caffeine]_0_ = 0.4 μM. Dashed lines represent the linear regressions for the five compounds.

Linear relationships were observed for the five compounds when plotting *k*_C,•OH_ against ln([C]_0_/[C]) throughout the treatments of the mixture of micropollutants with 50 μM NCl_3_ and 50 μM NHCl_2_ across the three pH values tested in this study (Figures 2 and S12). This reflects the critical role of •OH in degrading the five compounds in the NCl_3_–NHCl_2_ interactions. Unlike significant deviations in caffeine removal observed during breakpoint chlorination treatments at a Cl_2_/N ratio of 2, caffeine removals aligned well with the linear relationship, suggesting a minimal contribution of •ClO to its degradation. This is expected since •ClO produced from •OH scavenging by OCl^–^ was minimal under our experimental conditions, even at basic pH levels. Previous research suggested that the reaction of NCl_3_ with NHCl_2_ produces two HOCl/OCl^–^.^21,41^ However, in our experiments, background ammonia from the spiked NHCl_2_ solution would rapidly convert HOCl/OCl^–^ to NH_2_Cl, preventing •ClO formation from •OH scavenging by HOCl/OCl^–^. Indeed, experiments using a UV-spectrum-based approach to evaluate oxidant evolution and decomposition during the reaction of 50 μM NCl_3_ with 50 μM NHCl_2_ indicated less than 11 μM free chlorine throughout the experiments at pH 7 or 8.3 (Text S5).

While results from the breakpoint chlorination experiments suggest that an acidic pH favors the formation of •OH over nitrosating agents within the pH range of 5.8–7, we extended our research to study whether this pH dependency for the formation of nitrosating agents and •OH persists in the NCl_3_ reaction with NHCl_2_. A solution containing 10 μM Cl-DMA and 0.4 μM each of 1,4-dioxane and DEET at various pH values (5.8, 6.2, 6.6, and 7), buffered using 20 mM phosphates, was treated with 100 μM NCl_3_ and 40 μM NHCl_2_ for a predetermined reaction time at which the NCl_3_ concentration decomposed by approximately 90% (10 or 20 min; Figure S14b). The results show that increasing the pH from 5.8 to 7 increased NDMA formation concentrations at the expense of a slight decrease in micropollutant removal (Figure S14a), consistent with observations from breakpoint chlorination.

### Transient Reactive Species Analyses

**Figure 3:**
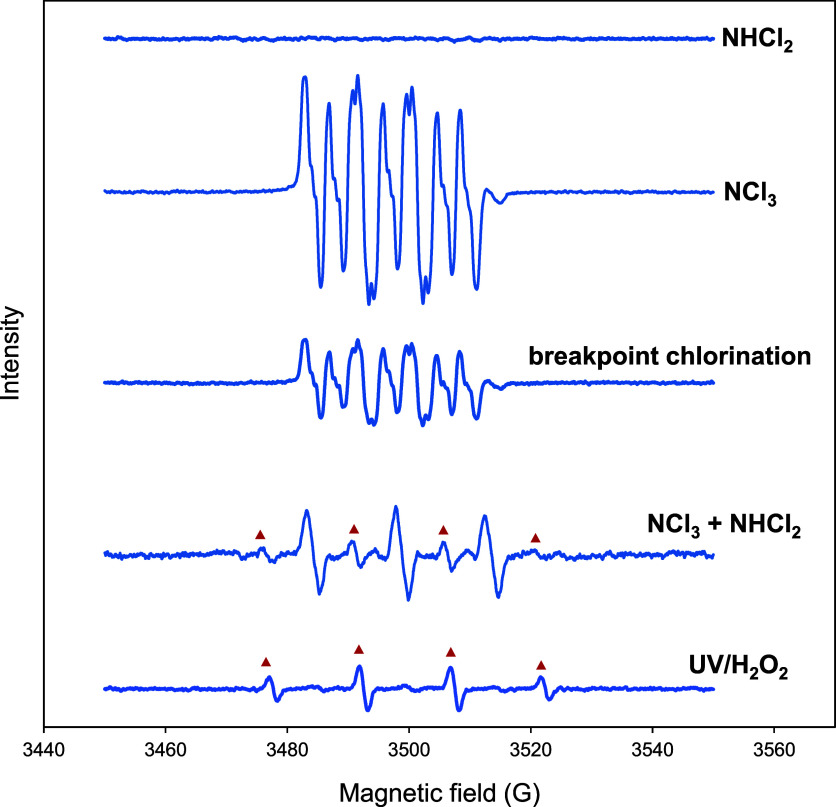
Electron spin resonance spectra of 80 mM DMPO in 20 mM phosphate buffer at pH 7 treated by 80 μM NHCl_2_ alone, 200 μM NCl_3_, breakpoint chlorination (480 μM HOCl + 240 μM NH_4_^+^), 200 μM NCl_3_ + 350 μM NHCl_2_, and UV (6W) + 6 mg/L H_2_O_2_ for 4 min (as a reference). Triangle symbols highlight the characteristic 1:2:2:1 resonance line for the DMPO–OH adduct. For treatment involving NCl_3_, a stoichiometric amount of H_2_O_2_ was added to quench the HOCl residual first.

The EPR spectra obtained from an 80 mM DMPO solution buffered at pH 7.0 (with 20 mM phosphates) subjected to treatment with NHCl_2_ alone, NCl_3_ alone (with H_2_O_2_ as the HOCl quencher), NCl_3_ + NHCl_2_, and breakpoint chlorination (480 μM HOCl + 240 μM NH_4_^+^), are presented in Figure 3. The DMPO solution treated with NHCl_2_ showed EPR silent, while the spectrum obtained from the DMPO solution treated by NCl_3_ alone closely resembled those treated with breakpoint chlorination. These resonance lines are consistent with previously reported spectra^6,42,43^ and can be attributed to 2-(*N*-chloroimino)-5,5-dimethylpyrrolidine-1-oxyl radical (ClDMPOX). Previous research has suggested that the formation of ClDMPOX is plausibly due to the reaction of DMPO with an intermediate containing an N–Cl functional group formed during breakpoint chlorination,^43^ and our results indicate it is attributed to NCl_3_.

The characteristic 1:2:2:1 resonance line for the DMPO–OH adduct was not detected in the spectrum recorded 2 min after treating an 80 mM DMPO solution with 200 μM NCl_3_ and 80 μM NHCl_2_. Instead, the obtained signals closely resembled those of the NCl_3_-treated DMPO solution. One possible explanation is that the resonance lines from ClDMPOX, formed by the interaction of NCl_3_ with DMPO, distorted those from the DMPO–OH adduct, thereby hindering the detection of •OH. Alternatively, the DMPO–OH adduct may be unstable in the presence of NCl_3_. To eliminate this interference, we prepared a sample by treating an 80 mM DMPO solution with 200 μM NCl_3_ and 350 μM NHCl_2_. The excess amount of NHCl_2_ relative to NCl_3_ facilitated the decomposition of NCl_3_. Consequently, the 200 μM NCl_3_ would decompose by over 99% within 50 s, leaving NH_2_Cl and NHCl_2_ in the reaction solution. Both NH_2_Cl and NHCl_2_ are EPR silent, ensuring clarity of the spectrum. The reaction mixture was immediately transferred to the EPR cell and placed in the spectrometer, and the spectrum was recorded. The spectrum highlighted the characteristic 1:2:2:1 signal. While these results suggest the formation of •OH during the reaction of NCl_3_ with NHCl_2_, we further validated •OH formation and explored potential mechanisms by using multiple probe compounds.

### Validating •OH Formation and Exploring Reaction Mechanisms with Probe Compounds

Since neither NCl_3_ nor NHCl_2_ contains oxygen, the •OH generated from their interaction must involve the participation of dissolved oxygen (O_2(aq)_) or H_2_O. However, experiments carried out to measure the change in O_2(aq)_ in the NCl_3_ reaction with NHCl_2_ indicated O_2(aq)_ remained unchanged before and after the reactions, even in experiments during which the NCl_3_ consumption (200 μM NCl_3_ consumed) was approximately equal to the initial O_2(aq)_ concentration (Text S6). Furthermore, micropollutant degradations in a N_2_-purged solution treated with NCl_3_ + NHCl_2_ showed no considerable difference from those in O_2_-saturated solutions (Figure S15). These results suggest negligible participation of O_2(aq)_ in the reaction between NCl_3_ and NHCl_2_.

Additional experiments tracing the origin of •OH oxygen during the NCl_3_–NHCl_2_ reaction revealed that terephthalate, a commonly used •OH probe, may produce misleading results due to the direct electron transfer reaction between NCl_3_ and terephthalate (see Text S7 for discussion). To avoid this issue, we employed benzene as a probe to trace the source of •OH oxygen by monitoring the source of oxygen in the phenol product in ^18^O-labeled H_2_O. Benzene was selected because it reacts highly with •OH (*k*_•OH_ = 7.8 × 10^9^ M^–1^ s^–1^)^44^ and forms phenol as the major product.^45^ Additionally, benzene’s oxidation potential is >0.7 V higher than oxygen-containing benzenes,^46^ making it less susceptible to direct electron transfer oxidation. Our control experiments showed that phenol formation was below the 0.1 μM detection limit when treating 100 μM benzene with 50 μM NCl_3_ for 20 min, with the benzene concentration remaining unchanged.

Treating 100 μM benzene in ^18^O–H_2_O by 50 μM NCl_3_ and 30 μM NHCl_2_ for 3 min produced ^18^O-phenol (quantified by *m*/*z* 96) at 0.31 μM, five times higher than that of ^16^O-phenol (0.06 μM; quantified by *m*/*z* 94). The ratio of ^18^O-phenol to total phenol was 84%, close to the expected 83% if water is the source of the oxygen in •OH, suggesting that H_2_O contributes to the oxygen source of •OH through the hydrolysis of intermediates formed in the NCl_3_–NHCl_2_ interactions.

Although N_2_ and NO_3_^–^ were suggested as the possible decomposition products from the reaction of NCl_3_ with NHCl_2_,^21,41,47^ no quantitative data were provided in these studies. To gain a deeper insight into the pathways for •OH formation in the reaction of NCl_3_ with NHCl_2_, we first evaluated the formation of nitrogen products during the reaction using ^15^N-isotopically labeled NCl_3_ and ^15^N-NHCl_2_. This evaluation helps to isolate the pathways for •OH formation from intricate cross-reactions during the NCl_3_–NHCl_2_ interactions. Figure S17a shows the concentrations of nitrogen species before and after the reaction of 75 μM ^15^N-NCl_3_ with 75 μM ^15^N-NHCl_2_ at pH 7, which contains 105 μM ammonia originating from NHCl_2_. Results indicated that NH_2_Cl and NHCl_2_ measured 20 min after the reactions were 77 ± 1.8 and 7.2 ± 0.1 μM, respectively. Ammonia measured in the thiosulfate-quenched samples was 100 ± 4 μM, equivalent to 39% of the 255 μM-N total initial nitrogen, while ^15^N–N_2_ formation was determined to be 174 ± 5 μM as N, corresponding to 68% of the 255 μM-N total initial nitrogen. NO_2_^–^ was below the 2 μM detection limit, and nitrate was 2.3 ± 0.7 μM. ^15^N-labeled N_2_O (i.e., ^15^N^15^NO) was barely detected (<1 μM as N). Repeating the experiments at pH 5.8 resulted in similar patterns for product formation; the major product ^15^N_2_ accounted for 52% of the total initial nitrogen. For these experiments, the sum of free ammonia, NH_2_Cl, NHCl_2_, N_2_, N_2_O, nitrite, and nitrate indicated nitrogen recoveries ranging from 94% to 109% in these experiments (Figure S17).

**Figure 4:**
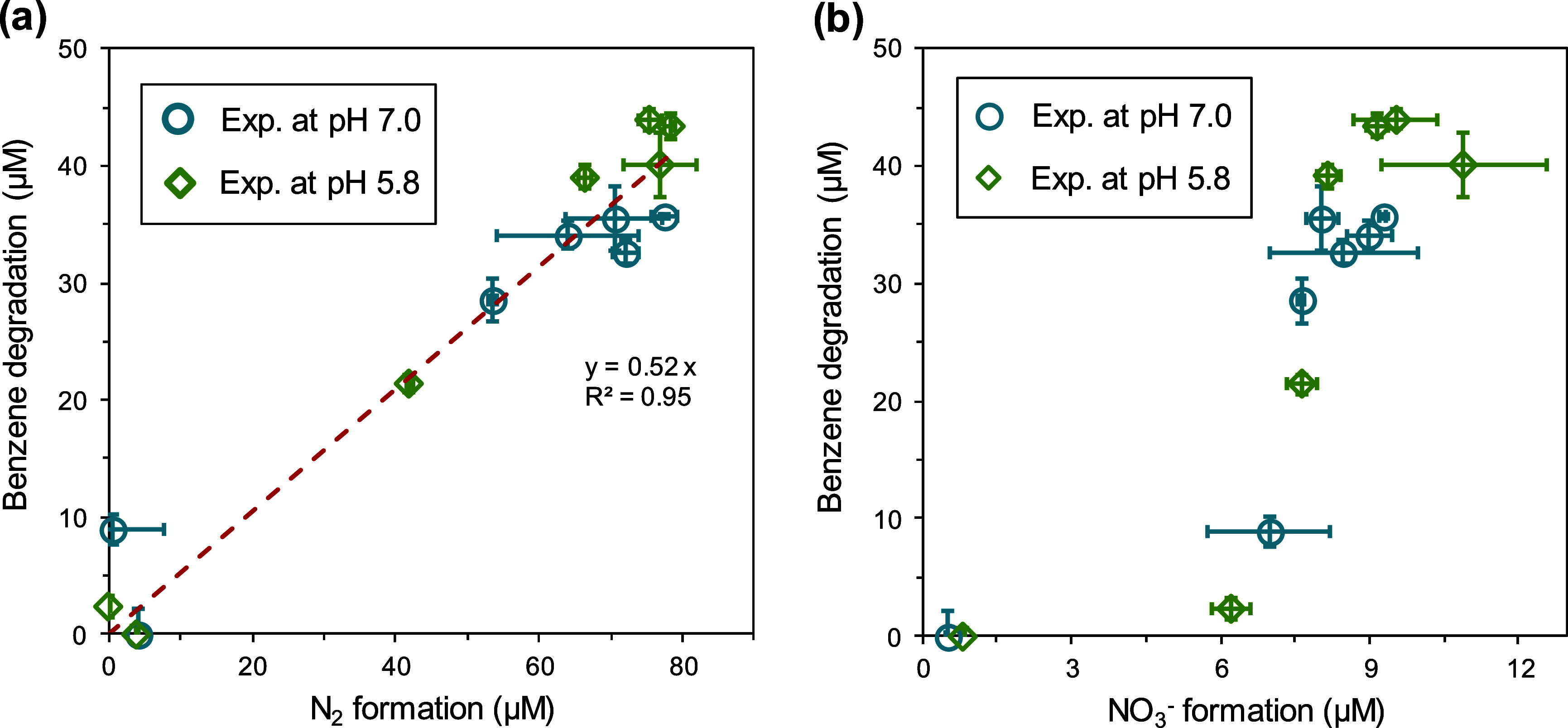
Relationship between benzene degradation and (a) N_2_ formation or (b) NO_3_^–^ formation during the treatments of 120 μM benzene in deionized water buffered at pH 7 or pH 5.8 (20 mM phosphates) by NCl_3_ + NHCl_2_ for 20 min. A total of 14 experiments were conducted, as detailed in Table S5.

Using this approach, we measured the formation concentrations of N_2_ and NO_3_^–^ in a total of 14 reactions and assessed which product is more closely associated with the generation of •OH. The experiments were carried out with ^15^N-NCl_3_ at 0 or 100 μM and ^15^N-NHCl_2_ varied from 0 to 100 μM at pH 5.8 and 7.0 (Table S5), and with 120 μM benzene to capture the •OH generated in the systems. Experiments at alkaline pH were omitted because of the rapid base-catalyzed self-decay of NCl_3_.^48^ While reactive nitrogen species may be generated in the NCl_3_–NHCl_2_ reaction, benzene remains inert toward those species (evaluated in this study, as detailed in Text S8), thereby making its degradation a surrogate for •OH formation. The results show a scattered relationship between the NO_3_^–^ formation and benzene decomposition (Figure 4). By contrast, a linear relationship between N_2_ formation and benzene decomposition was highlighted across both pH levels (5.8 and 7.0), with one mole of benzene degradation corresponding to the formation of 1.92 mol of N_2_ (Figure 4). This result suggests that •OH formation is attributed to competing reactions among complex branching pathways through which N_2_ is generated. Although not explicitly elucidated, we propose that the formation of 1,1-dichlorodiazine may serve as a key branching point with H_2_O nucleophilic attack on the =N:^+^ moiety to form a hydroxyl diazine intermediate (Reaction R5 in Scheme 1). This hydroxyl diazine structure has been reported to be capable of undergoing homolytic fission, leading to •OH, •Cl, and N_2_ among others, in a fashion similar to those occurred in HO—N=N—OH that result in two •OH and a N_2_.^17,18^ The formation of •Cl from homolytic fission is possible yet was not confirmed in this study. However, if •Cl were produced, it would quickly convert to •OH through its reaction with water (i.e., •Cl + H_2_O → H^+^ + •ClOH^–^ (*k* = 2.5 × 10^5^ s^–1^) and •ClOH^–^ → •OH + Cl^–^).^49^ The branch ratio for Reaction R5 over Reaction R4 would be 52%/48%, leading to one mole of •OH formation accompanied by 1.92 mol of N_2_ formed. While the proposed pathways aligned with the •OH oxygen source tracing results, future research is needed to fully elucidate the mechanisms.

Phenol formation was monitored and generally matched the trends for micropollutant removals (Figures S19 and S20). Phenol yields relative to benzene loss ranged from 0 to 17 ± 0.1%, significantly lower than the 45 ± 3% reported for Fe^2+^/H_2_O_2_ treatment of benzene.^50^ If •OH were the main oxidant driving phenol formation in NCl_3_–NHCl_2_ reactions, we would expect yields that are consistent and comparable to the 45 ± 3% previously reported in the literature. Thus, the results suggest that system oxidants may influence phenol formation by altering the reaction pathways or causing further decomposition of phenol.

Formation of phenol from the reaction of •OH with benzene involves multiple steps. •OH adds to benzene to form a hydroxycyclohexadienyl radical (C_6_H_6_OH•) (R9) with a near-diffusion-controlled rate constant (*k* = 7.9 × 10^9^ M^–1^ s^–1^). C_6_H_6_OH• undergoes reversible O_2_ addition to generate hydroxycyclohexadienylperoxyl radicals (C_6_H_6_OOOH•; *k* = 3.9 × 10^8^ M^–1^ s^–1^) (R10). C_6_H_6_OOOH• decomposes to phenol and HO_2_• (R11), among other products (R12). Moreover, phenolate (R13) formed during the reaction of NCl_3_ with NHCl_2_ in the presence of benzene may decompose via reactions with NCl_3_ (R14) or HOCl (R15).^40,51^
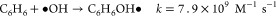
R9
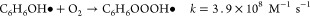
R10
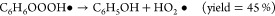
R11
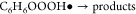
R12
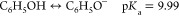
R13
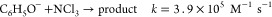
R14
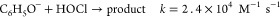
R15

We used a kinetic model to assess the extent to which these •OH-associated reactions (i.e., R9–R15) explain phenol formation during NCl_3_–NHCl_2_ interactions. This model is based on the widely used UF model, with modifications including redetermined rate constants for the reactions between NCl_3_ and NHCl_2_ and between NCl_3_ and NH_2_Cl at pH 7. Additionally, it incorporates an extra branch reaction that elucidates the formation of a nitrosating agent (No. 16 in Table S6).^11^ Our recent study demonstrated that this kinetic model successfully predicts the evolution and decomposition of oxidants during the reaction of NCl_3_ with NHCl_2_, as well as during breakpoint chlorination.^11^

Since branch reactions exist in the reactions between NCl_3_ and NHCl_2_ through which N_2_ forms as an ultimate product, we revised this reaction (NCl_3_ + NHCl_2_ → 2 HOCl + N_2_) to NCl_3_ + NHCl_2_ → 2 HOCl + N_2_ + 0.52 •OH. The stoichiometry of the revised reaction reflects the observation that 1.92 mol of N_2_ formation per mole of benzene degraded (Figure 4). The radical scavenging reactions by oxidants and key parameters adapted from the literature (e.g., the rate constants and the phenol yield relative to benzene loss) were combined within the chemical kinetic model (Table S6) to simulate benzene loss and formation of products (detailed in Text S9). For mixtures of 120 μM benzene in 20 mM buffered deionized water at pH 7 treated with 100 μM NCl_3_ and 0–100 μM NHCl_2_ (i.e., Exp. Nos. 7–13 in Table S5), the experimental losses of benzene and formation of phenol matched with the model predictions quite well (within 20% error; Figure S21a), reflecting the critical role of •OH formed from the NCl_3_–NHCl_2_ interaction in transforming benzene to phenol. Modeling also predicted N_2_ formation well (Figure S21a).

We also used *tert*-butanol alongside benzene to assess •OH formation. *Tert*-butanol primarily produces formaldehyde via an alkyl radical (•CH_2_C(CH_3_)_2_OH) intermediate formed through H-abstraction from the −CH_3_ group, with the rate constants and product yields for the key reactions well-established in the literature (Text S10).^32,52^ Overall, our experimentally measured formaldehyde concentrations closely matched model predictions across various experiments, further confirming •OH generation during NCl_3_–NHCl_2_ interactions (details are provided in Text S10).

### The Importance of •OH Formation via NCl_3_–NHCl_2_ Reactions during Breakpoint Chlorination: Kinetic Modeling

**Figure 5:**
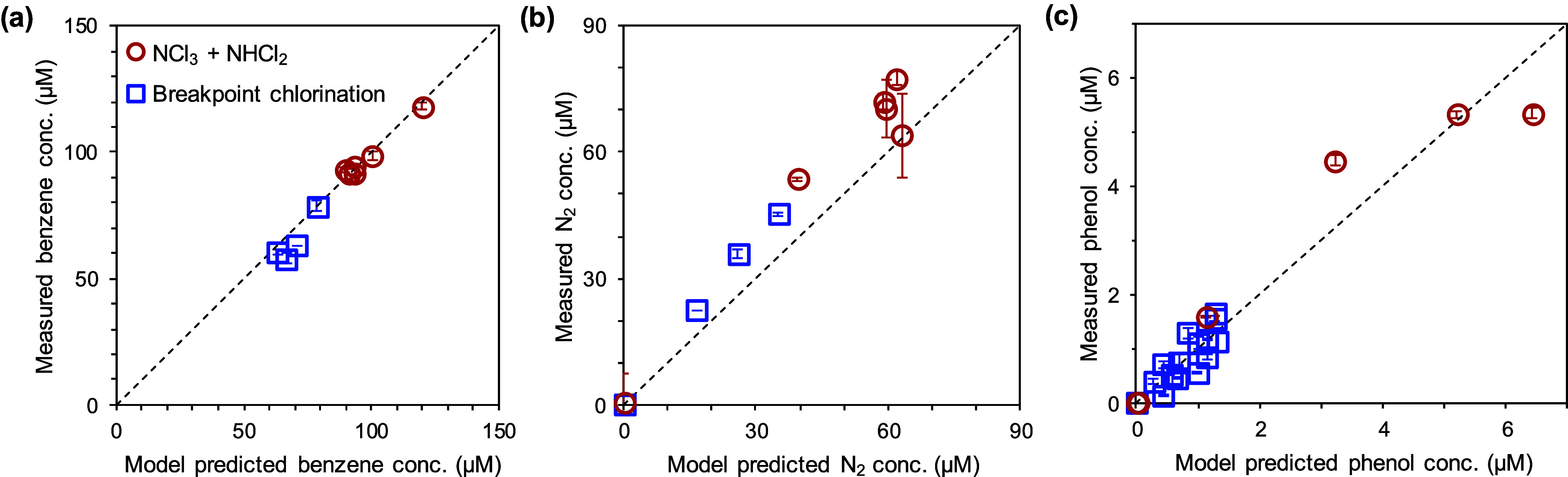
Comparison of model-predicted versus measured concentrations in all experiments, including NCl_3_–NHCl_2_ treatments and breakpoint chlorination at pH 7.0. Lines indicate the 1:1 ratio. Error bars represent the ranges from the experimental duplicates.

We adapted this kinetic model to evaluate the significance of NCl_3_–NHCl_2_ interactions in •OH formation during breakpoint chlorination. Four additional experiments were conducted to measure benzene loss and ^15^N–N_2_ formation at 30 min after employing HOCl and ^15^N-NH_4_^+^ at different concentrations to treat 80 μM benzene (Exp. Nos. 15–18 in Table S5). Additionally, phenol concentrations were measured over time in two of those experiments (Exp. Nos. 16 and 17 in Table S5). Overall, the model well predicted N_2_ formation, benzene loss, and the trends for phenol formation within a 28% error (Figure S21b,c); phenol formation spiked within the first 5 min followed by downtrends (Figure S21c), driven by reacting with HOCl and NCl_3_ (Reactions R14 and R15). Figure 5 compares the model-predicted versus measured concentrations in all of the experiments, including NCl_3_–NHCl_2_ treatments and breakpoint chlorination (as listed in Table S5). The good alignments between model-predicted values and experimental data showcase that •OH formation in breakpoint chlorination can be attributable to the NCl_3_–NHCl_2_ interaction.

### Environmental Implications

Breakpoint chlorination reactions are commonly observed during disinfection in various scenarios, including drinking water treatment, potable reuse processes, and swimming pool water disinfection. One of the examples is the chlorination of the effluents from full-advanced treatment (FAT) trains for potable reuse. Chlorination of FAT effluents with upstream chloramines antifoulant^53^ initiates breakpoint reactions. This process generates •OH and other reactive species that may provide additional chemical barriers for micropollutant removal. However, it also enhances the formation of harmful nitrosamine carcinogens. Understanding the underlying mechanisms and dual nature of breakpoint chlorination is crucial to optimizing water treatment processes.

Our study demonstrated that the formation of nitrosation agents and •OH in breakpoint chlorination results from the interaction between NCl_3_ and NHCl_2_, through which parallel reactions occur to generate N_2_ alongside •OH. Two key factors influencing the trade-offs between nitrosamine formation and micropollutant removal are pH and the Cl_2_/N ratio. Acidic pH favors •OH formation over nitrosating agents. Despite the slower overall reaction kinetics for breakpoint reactions at acidic pH, which require approximately 30 min to complete, the longer contact time in a typical disinfection basin (e.g., 90 min)^54^ ensures the completion of breakpoint chlorination and maximizes •OH exposure (i.e., ∫[•OH]d*t*). Therefore, a slightly acidic condition (e.g., the effluent of FAT; pH = ∼5.5–6) for chlorination may be beneficial for micropollutant removal and nitrosamine control.

The Cl_2_/N molar ratio significantly affects the overall •OH exposure in breakpoint chlorination and, consequently, micropollutant removal. This relationship can be quantitatively rationalized by using the kinetic model developed in this study. Kinetic modeling incorporating the *k*_•OH_ for micropollutants accurately predicted micropollutant removal across Cl_2_/N ratios from 0 to 3 (Figure S23). These findings further emphasize the critical role of NCl_3_–NHCl_2_ interactions in •OH formation, particularly in breakpoint chlorination with a Cl_2_/N ratio of ≥1.5. When implementing the kinetic model to simulate •OH exposure and cumulative •ClO formation within the first 30 min of breakpoint chlorination, results show a drastic drop in •OH exposure after peaking at Cl_2_/N = 2, at the expense of increasing cumulative •ClO formation (Figure S24a). This concurs with the trends of micropollutant removals, which maximize at a Cl_2_/N of 1.8–2 and decrease afterward (Figure S6). Maintaining a chlorine dosage to achieve a Cl_2_/N ratio of 2 in the disinfection unit would achieve maximal micropollutant removal, although this is less practically feasible. However, due to the high reactivity of •ClO with aromatics bearing electron-donating groups,^55^ water utilities employing chlorine at high Cl_2_/N ratios can harness the oxidation power of •ClO to remove a broad array of micropollutants while constraining nitrosamine formation. Indeed, our experiments in 10 mM buffered deionized water at pH 7 and in authentic drinking water, using 1,4-dimethoxybenzene (*k*_•ClO_ = 2.1 × 10^9^ M^–1^ s^–1^)^56^ and carbamazepine (*k*_•ClO_ = 2.0 × 10^8^ M^–1^ s^–1^)^57^ at 0.5 μM each, showed consistent (70–100%) removal of both compounds as the Cl_2_/N molar ratio exceeded 2.5 (Figure S24b,c).

## Supporting Information Available

Chemical lists, analytical methods for gaseous and ionic nitrogen species, figures for micropollutant removals during the treatments of breakpoint chlorination or NCl_3_–NHCl_2_ interactions, control experiments for the reactivities of micropollutants with oxidants, •OH oxygen source tracing method, kinetic model development, and additional experimental results and discussion (PDF)
