# Supplementary material for: Unveiling the Critical Pathways of Hydroxyl Radical Formation in Breakpoint Chlorination: The Role of Trichloramine and Dichloramine Interactions
Source: Environ Sci Technol. 2024 Nov 11;58(47):21086–96. doi: 10.1021/acs.est.4c08403 (PMC11603780; doi:10.1021/acs.est.4c08403)
Supplement: Supplementary file 1 — es4c08403_si_001.pdf [file es4c08403_si_001.pdf]

Supporting Information

for

Unveiling the Critical Pathways of Hydroxyl Radical Formation in Breakpoint Chlorination: The Role of Trichloramine and Dichloramine Interactions

Yi-Hsueh Chuang*, Chia-Shun Chou, Yi-Lin Chu

Institute of Environmental Engineering, National Yang Ming Chiao Tung University

1001, University Rd., Hsinchu city, Taiwan 30010

*Corresponding author: yhchuang@nycu.edu.tw

This supporting information contains:

32 pages

10 Texts

24 Figures

6 Tables

1 Scheme

Text S1. Additional discussion for the formation of nitrosamines and degradations of micropollutants during the breakpoint chlorination treatments.

Separate studies on the degradation of HOCl-resistant compounds, which indicate the quantity of reactive species generated, have demonstrated that micropollutant degradation behavior parallels that of nitrosamine formation. For instance, Ye et al.[1] evaluated the degradations of atrazine, benzotriazole, and other compounds during the chlorination of ammonia-containing waters at Cl_2/N molar ratio ranging from 0–4. Patton et al.[2] evaluated the degradation of 1,4-dioxane in breakpoint chlorination with Cl_2/N molar ratio ranging from 0–3. Their results clearly indicated that those micropollutants' removals exhibited volcano-shaped trends with Cl_2/N ratios ranging from 0 to 4 at neutral pH (Figure S1); the removal rates increased as the Cl_2/N molar ratio rose from 0 to approximately 1.5–2, then declined afterwards. This pattern aligns with observations of nitrosamine formation during the chlorination of ammonia-containing water with dimethylamine (DMA) precursor.[3]

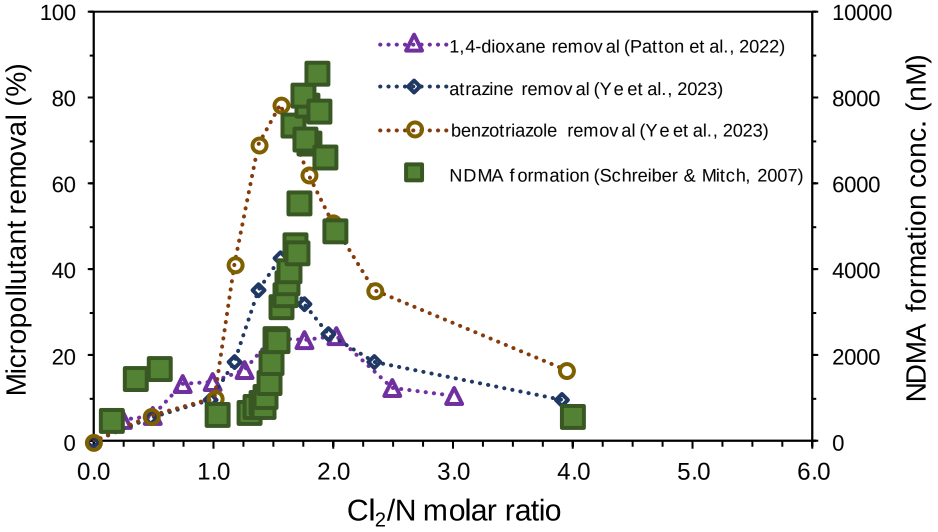

Figure S1. Plot-digitized data of NDMA formation concentration measured 6 h after treating a mixture of 10 μM dimethylamine and 120 μM NH_4^+ with 5−500 μM HOCl at pH 6.9 (10 mM phosphate buffer), and plot-digitized data of micropollutant removals (initial concentrations = 20 µg/L) at 20 min after the treatment of 2 mg-N/L by HOCl at pH 7.0. Data were obtained from the Ye et al.[1], Patton et al.[2], and Schreiber and Mitch.[3]

In another study, Wang et al.[4] evaluated the removals of carbamazepine during the treatment of a mixture of 205 μM NH_4^+ and 8.5 μM carbamazepine by 563 μM Cl_2 at pH 5.5, 7.0, and 9.5. Their results showed a linear relationship between carbamazepine removals and total chlorine losses across all pH tested. Chuang et al.[5] examined the formation of NDMA when a mixture of 7.5 μM *N*-chloro-dimethylamine and 50 μM NH_4^+ was treated by 100 μM HOCl at pH 7, and reported a linear relationship between NDMA formation and total chlorine loss. Those plot-digitized data were shown in Figure S2. Micropollutant removals linearly correlated with the loss of total chlorine in breakpoint chlorination,[4] aligning the patterns for nitrosamine formation.[5]

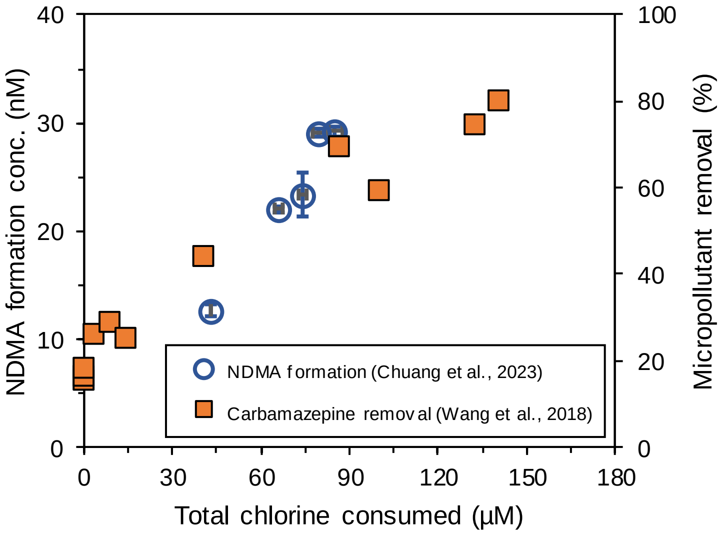

Figure S2. Relationship between carbamazepine removals and total chlorine consumed during the treatment of a mixture of 205 µM NH_4^+ and 8.5 µM carbamazepine by 563 µM Cl_2 at pH 5.5, 7.0, and 9.5. Additionally, the relationship between NDMA formation concentrations and total chlorine consumed during the treatment of a mixture of 7.5 µM *N*-chloro-dimethylamine and 50 µM NH_4^+ with 100 µM HOCl at pH 7 was presented. Data were digitized from plots by Chuang et al. and Wang et al.[4, 5]

Table S1. The unified (UF) model developed by Jafvert and Valentine.[6]

No.	Reaction	Rate expression	Rate constant for the k_U (at 25 °C)
U1	$HOCl + NH_3 \rightarrow NH_2Cl + H_2O$	$k_{U1}[HOCl][NH_3]$	4.17×10^{6} $M^{-1}s^{-1}$
U2	$NH_2Cl + H_2O \rightarrow HOCl + NH_3$	$k_{U2}[NH_2Cl]$	2.10×10^{-5} s^{-1}
U3	$NH_2Cl + HOCl \rightarrow NHCl_2 + H_2O$	$k_{U3}[HOCl][NH_2Cl]$	2.78×10^{2} $M^{-1}s^{-1}$
U4	$NHCl_2 + H_2O \rightarrow NH_2Cl + HOCl$	$k_{U4}[NHCl_2]$	6.39×10^{-7} s^{-1}
U5	$NH_2Cl + NH_2Cl \rightarrow NHCl_2 + NH_3$	$k_{U5}[NHCl_2]^2$	6.94×10^{3} $M^{-2}s^{-1}$ (a)
U6	$NHCl_2 + NH_3 + H^+ \rightarrow NH_2Cl + NH_2Cl + H^+$	$k_{U6}[NH_3][NHCl_2][H^+]$	6.00×10^{4} $M^{-2}s^{-1}$
U7	$NHCl_2 + H_2O \rightarrow$ *I* (HNO) + products	$k_{U7}[NHCl_2][OH^-]$	1.10×10^{2} $M^{-1}s^{-1}$
U8	$NHCl_2$ + *I* (HNO) $\rightarrow$ HOCl + products	$k_{U8}[NHCl_2][I]$	2.78×10^{4} $M^{-1}s^{-1}$
U9	NH_2Cl + *I* (HNO) $\rightarrow$ products	$k_{U9}[NH_2Cl][I]$	8.30×10^{3} $M^{-1}s^{-1}$
U10	$NH_2Cl + NHCl_2 \rightarrow$ products	$k_{U10}[NH_2Cl][NHCl_2]$	1.53×10^{-2} $M^{-1}s^{-1}$
U11	$NHCl_2 + HOCl \rightarrow NCl_3 + H_2O$	$k_{U11}[NHCl_2][HOCl]$	(b)
U12	$NCl_3 + NHCl_2 \rightarrow HOCl + HOCl$ + products	$k_{U12}[NCl_3][NHCl_2]$	5.56×10^{3} $M^{-1}s^{-1}$ (c)
U13	$NCl_3 + NH_2Cl \rightarrow HOCl$ + products	$k_{U13}[NCl_3][NH_2Cl]$	1.39×10^{2} $M^{-1}s^{-1}$ (d)
U14	$2HOCl + NHCl_2 + H_2O \rightarrow NO_3^- + 5H^+ + 4Cl^-$	$k_{U5}[HOCl][NHCl_2]$	2.30×10^{2} $M^{-1}s^{-1}$

(a) $k_{U5} = k_H[H^+] + k_{H2PO4-}[H_2PO_4^-] + k_{H3PO4}[H_3PO_4] + k_{H2CO3}[H_2CO_3] + k_{HCO3-}[HCO_3^-]$.
where $k_H = 6.9\times10^{3}$ $M^{-2}s^{-1}$, $k_{H2PO4-} = 3.6\times10^{-1}$ $M^{-2}s^{-1}$, $k_{H3PO4} = 8.9\times10^{2}$ $M^{-2}s^{-1}$, $k_{H2CO3} = 7.5\times10^{-1}$ $M^{-2}s^{-1}$, $k_{HCO3-} = 2.0\times10^{-3}$ $M^{-2}s^{-1}$. k_{U5} is 6.94×10^{3} $M^{-2}s^{-1}$ at pH 7.

(b) $k_{U11} = k_{OCl-}[OCl^-] + k_{HPO42-}[HPO_4^{2-}] + k_{OH-}[OH^-]$.
where $k_{OCl-} = 9\times10^{4}$ $M^{-2}s^{-1}$, $k_{HPO42-} = 1.6\times10^{4}$ $M^{-2}s^{-1}$, $k_{OH-} = 3.3\times10^{9}$ $M^{-2}s^{-1}$

(c) the kinetic expression for U12 was $k_{U12'}[NCl_3][NHCl_2][OH^-]$, where $k_{U12'}$ was 5.6×10^{10} $M^{-2}s^{-1}$. At pH 7, $k_{U12} = k_{U12'}[OH^-] = 5.6\times10^{3}$ $M^{-1}s^{-1}$.

(d) the kinetic expression for U13 was $k_{U13'}[NCl_3][NH_2Cl][OH^-]$, where $k_{U13'}$ was 1.4×10^{9} $M^{-2}s^{-1}$. At pH 7, $k_{U13} = k_{U13'}[OH^-] = 1.4\times10^{2}$ $M^{-1}s^{-1}$.

Table S2. Chemicals.

Chemical	Concentration or purity	Supplier
Dimethylamine	40 wt.%	Acros Organics
EPA 521 Nitrosamine Mix	2000 µg/mL each component in dichloromethane	Sigma-Aldrich
EPA521 internal standard	1000 µg/L in Methylene Chloride	Accustandard
Dichloromethane	99.9%	Honeywell
Sodium hypochlorite	(min.6%)	Duksan
Ammonium chloride	ACS grade	Macron
Sodium hydroxide	≥98%	Honeywell
Potassium phosphate monobasic	>99%	Acros Organics
Sodium sulfite	≥98%	Acros Organics
Sodium chloride	99.5%	Honeywell
Orthophosphoric acid	85%	Fisher Scientific
Sodium nitrite	98%	Alfa Aesar
Sodium nitrate	≥98%	Alfa Aesar
Ascorbic acid	Reagent grade	Sigma-Aldrich
Potassium iodide	>99.5%	Honeywell Fluka
DPD Oxalate N,N-Diethyl-p-Phenylenediamine	Analytical Grade	Hach
Hydrogen peroxide	34.5%-36.5%	Honeywell
1,4-dioxane	99.8%	Thermo scientific
DEET	98%	Acros Organics
Caffeine	99%	Alfa Aesar
Nitrobenzene	99%	Thermo Scientific
Benzoic acid	99%	Thermo Scientific
Carbamazepine	>97%	TCI
1,4-dimethoxylbenzene	99+%	Acros Organics
Benzene	≥99.7%	Sigma-Aldrich
Benzene-d6	99.5%	Aladdin
$^{18}O-H_2O$	97% atom	Aladdin
$^{15}N-NH_4Cl$	≥98%	Sigma-Aldrich
$^{15}N-N_2$	98%	Sigma-Aldrich
Terephthalic acid	99+%	Acros Organics
Formaldehyde solution	36.5–38%	Sigma-Aldrich
2,4-dinitrophenylhydrazine	98%	Macklin

Table S3. Summary of the rate constants of micropollutants with radicals.

Micropollutant	$k_{\bullet OH}$ ($\times10^9$ $M^{-1}s^{-1}$)	$k_{\bullet Cl}$ ($\times10^9$ $M^{-1}s^{-1}$)	$k_{\bullet ClO}$ ($\times10^8$ $M^{-1}s^{-1}$)	$k_{\bullet Cl2-}$ ($\times10^6$ $M^{-1}s^{-1}$)	RNS[a] ($M^{-1}s^{-1}$)	References
1,4-Dioxane	2.5–3.2	0.0044	NA	<0.05–3.3	NA	[7-9]
Nitrobenzene	3.2–4.7	0.52	0.002	<0.5	Negligible	[8, 10, 11]
Benzoate	5.5–6.2	14	<0.03	<2	Negligible	[10-13]
Caffeine	6.4	14.6–38.7	1.03	9.28×10^8	NA	[12, 14, 15]
DEET	5.0–7.5	3.8	slow	NA	$\leq1\times10^9$	[11, 14, 16, 17]

[a] RNS may include $\bullet NO$, $\bullet NO_2$, $\bullet NH_2$. NA = not available.

Text S2. Analyses for the formation of N_2 (^{15}N-N_2), N_2O (^{15}N-N_2O), and inorganic nitrogen species during ^{15}N-NCl_3 reaction with ^{15}N-$NHCl_2$

Gaseous and ionic nitrogenous products were quantified in the breakpoint chlorination or in the reaction of NCl_3 with $NHCl_2$. To initiate an experiment, four 22-mL glass vial was filled with 11-mL deionized water, buffered at desired pH with 20 mM phosphates containing ^{15}N isotopically labeled NCl_3 (with stoichiometric amount of H_2O_2 to quench the HOCl residual first). The vials were immediately sealed with a solid-PTFE-septa inserted open-top cap after spiking ^{15}N-$NHCl_2$ at target concentration. After 20 minutes of reaction time, while two of the four vials were sacrificed for analysis of NH_2Cl and $NHCl_2$ residuals using the DPD colorimetric method,[18] the other two sample vials were spiked with a small aliquot of thiosulfate to quench the chlorine residuals using a microsyringe. Sample vials were kept at room temperature (25±1 °C), vigorously shaken for 1 min, and allowed to stand for 30 min prior to sampling; these approaches facilitate the equilibrium of volatile nitrogen products (N_2, N_2O) between gaseous phase and water phase. An aliquot (5 μL) of the headspace sample was taken using a microsyringe, and was injected into an Agilent 7890N GC equipped with an RTX-200 column and an Agilent 5977A mass spectrometer. The background argon (m/z 40; abundance = 0.934% or 9340 ppmv in ambient air) serves as an internal standard for N_2O quantification. Analytes were quantified under electron impact ionization in single ion monitoring mode (m/z of 28 for ^{14}N-N_2, m/z 30 for ^{15}N-N_2, m/z 46 for ^{15}N-N_2O, and m/z 40 for Ar). The remaining solutions were analyzed for the concentrations of ammonia, nitrate, and nitrite. Note that ammonia concentrations measured in the samples quenched with thiosulfate encompass inorganic chloramines (i.e., NH_2Cl, $NHCl_2$, NCl_3), which were reduced to ammonia by reacting with the thiosulfate quencher.

The ratio of ^{15}N-N_2/^{14}N-N_2 in the gaseous sample was calculated based on the area counts for m/z 30 and m/z 28. The partial pressure of ^{15}N-N_2 was determined by multiplying the partial pressure of ^{14}N-N_2 (i.e., 0.78 atm) by the ratio of ^{15}N-N_2/^{14}N-N_2. A Sigma-Aldrich ^{15}N-N_2 (98 atom % ^{15}N) standard was used to prepare a standard curve, as shown in Figure S3a. Results from injecting ambient air as a control showed that signals for m/z 30 were negligible. A Henry's constant of 6.4×10^{-6} mol/m^3/Pa for N_2 at 25 °C was used to calculated the concentration of dissolved N_2 in the solution.[19]

The formation of ^{15}N-N_2 during the reaction of ^{15}N-NCl_3 with ^{15}N-$NHCl_2$ was calculated by dividing the ^{15}N-N_2 measured in the gas phase and the ^{15}N-N_2 in solution by the volume of the solution (Eq. S1). In Eq. S1, $[^{15}N\text{-}N_2]_{(g)}$ denotes the concentration of ^{15}N-N_2 measured in the gas phase, and V_g is the volume of the gas phase (11-mL in our case). While $[^{15}N\text{-}N_2]_{(aq)}$ denotes ^{15}N-N_2 concentration in the solution, calculated using the 6.4×10^{-6} mol/m^3/Pa Henry's constant, V_s is the volume of solution. Because one molecule of N_2 contains two nitrogen atoms, a coefficient of 2 is incorporated into the equation.

$$[^{15}N\text{-}N_2] \text{ formed (μM as N)} = ([^{15}N\text{-}N_2]_{(g)} \times V_g + [^{15}N\text{-}N_2]_{(aq)} \times V_s) \times 2 / V_s \qquad \text{(Eq. S1)}$$

To quantify ^{15}N-N_2O, we monitored the area ratio of m/z 46 to m/z 40. Since ^{15}N-N_2O is not commercially available, a series of standard samples were prepared by diluting a 5000-ppmv ^{14}N-N_2O (Chiah Lung Enterprise Co., Ltd, Taiwan) with high-purity helium in 22-mL vials, with Argon gas (Sigma-Aldrich 99.9%) spiked at 9340 ppmv as the internal standard. Figure S3b illustrates the results. ^{14}N-N_2O exhibits an m/z of 44 in the mass spectrum, which overlaps with the m/z 44 from ambient CO_2. Consequently, an intercept was detectable in the standard curve. Nevertheless, the slope obtained from the regression of the (m/z 44)/(m/z 40) ratio against N_2O concentration can be utilized to quantify the concentration of ^{15}N-N_2O produced in the experiments, assuming the response of ^{15}N-N_2O in the mass spectrometer is the same with that of ^{14}N-N_2O. A Henry's constant of 2.4×10^{-4} mol/m^3/Pa for N_2O at 25 °C was used to calculated the concentration of dissolved N_2O in the solution.[19] Results from injecting ambient air as a control showed that signals for m/z 46 were negligible.

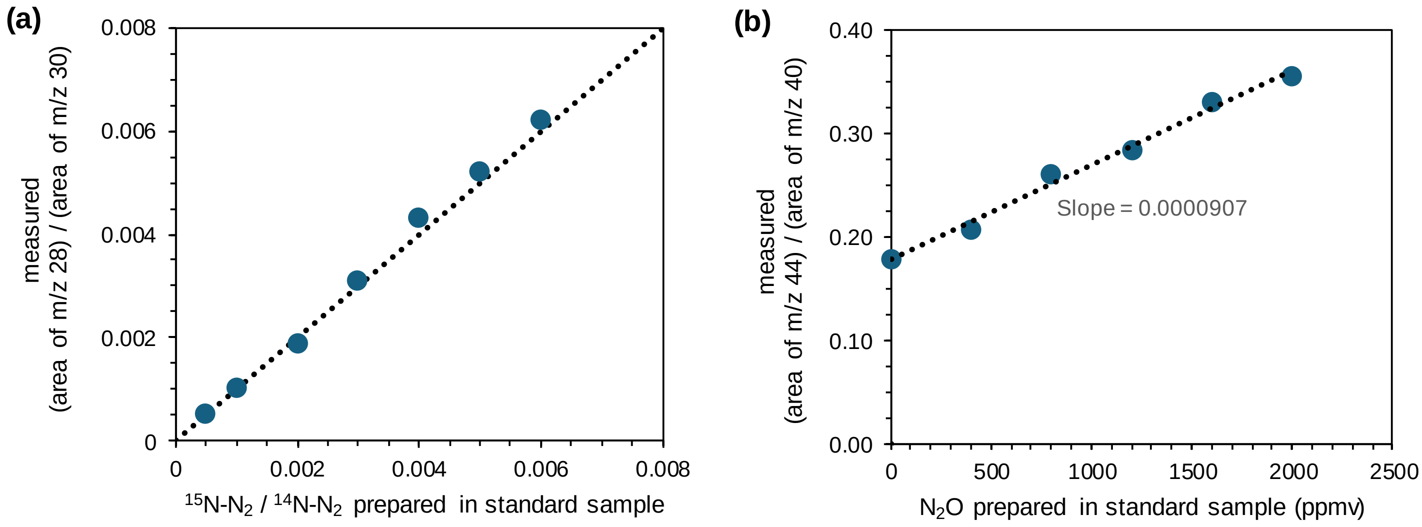

Figure S3. (a) ^{15}N-N_2/^{14}N-N_2 prepared in standard samples versus the measured area ratios between m/z 28 and m/z 30. Dashed line represents 1:1 ratio. (b) N_2O standard curve from a series of standard samples containing 0–2000 ppmv N_2O in helium with 9340 ppmv Ar. Dashed line represents the linear regression.

Text S3. Analytical method for benzene

In this study, experiments were conducted to quantify the formation of •OH by measuring the loss of benzene and the formation of phenol during the treatment of benzene by NCl_3 + $NHCl_2$ or by breakpoint chlorination (see the section '**Validating •OH formation and exploring reaction mechanisms with probe compounds.**' in the main text). To initiate an experiment, a 22-mL vial containing 10-mL phosphate buffer at desired pH and 100 µM benzene was dosed with NCl_3 (with stoichiometric amount of H_2O_2 to quench the HOCl first) and $NHCl_2$ at target concentrations (or HOCl and NH_4^+ at the target concentrations). Note that the vial was immediately capped after spiking the oxidants. A microsyringe was used to injected $Na_2S_2O_3$ quencher (500 µM) into the solution to terminate the reaction after certain reaction time, followed by the injection of benzene-d6 surrogate (100 µM). Then, the sample vial was kept at room temperature (25±1 °C), vigorously shaken for 1 min, and allowed to stand for 30 min prior to sampling; these approaches facilitate the equilibrium of volatile analytes (e.g., benzene, N_2, N_2O) between gaseous phase and water phase. An aliquot (5 µL) of the headspace sample was taken using a microsyringe, and was injected into an Agilent 7890N GC equipped with an RTX-200 column and an Agilent 5977A mass spectrometer.

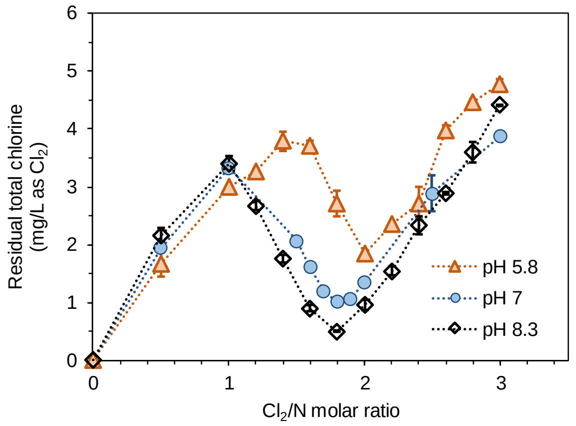

Figure S4. Total chlorine concentrations measured at 60 min after employing 0–150 μM HOCl into a solution of 50 μM NH_4^+ at deionized water buffered at pH 5.8, 7, or 8.3 (10 mM phosphates). Error bar represents the range of duplicate experiments.

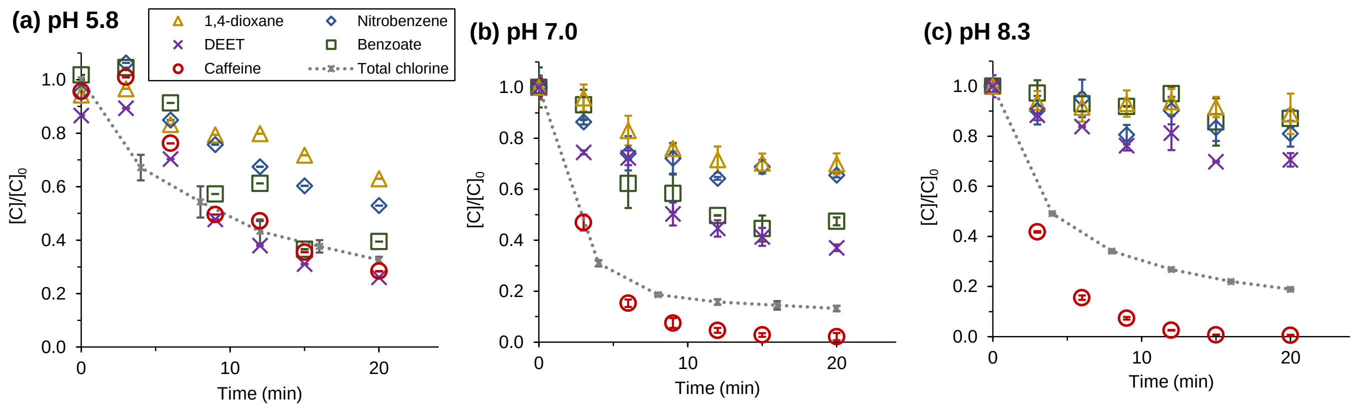

Figure S5. Concentrations of total chlorine and micropollutants during the treatment of a mixture of micropollutants at 0.2 or 0.4 μM each and 50 μM NH_4^+ by 100 μM HOCl at (a) pH 5.8, (b) pH 7.0, and (c) pH 8.3. The pH of the solutions was maintained by 10 mM phosphates. ($[1,4\text{-dioxane}]_0 = [DEET]_0 = 0.2$ μM, $[benzoate]_0 = [nitrobenzene]_0 = [caffeine]_0 = 0.4$ μM). Error bar represents the range of duplicate experiments.

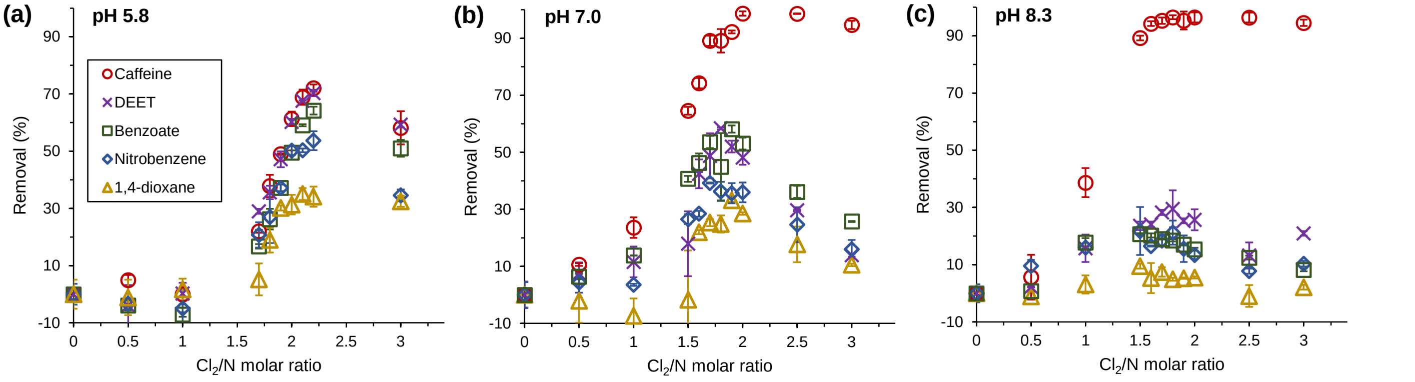

Figure S6. Micropollutants removals measured at 20 min after treating a mixture of 0.2 or 0.4 μM micropollutants and 50 μM NH_4^+ by various concentration of HOCl (0–150 μM) at (a) pH 5.8, (b) pH 7.0, and (c) pH 8.3. The pH of the solutions was maintained by 10 mM phosphates. ($[1,4\text{-dioxane}]_0 = [\text{DEET}]_0 = 0.2$ μM, $[\text{benzoate}]_0 = [\text{nitrobenzene}]_0 = [\text{caffeine}]_0 = 0.4$ μM). Error bar represents the range of duplicate experiments.

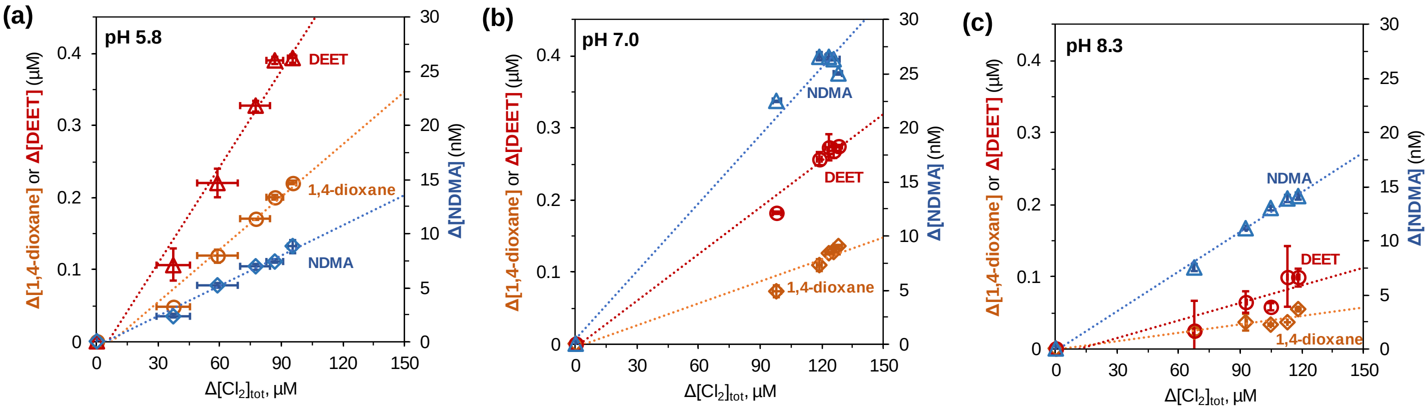

Figure S7. Relationships among the change of total chlorine concentration ($\Delta[Cl_2]_{tot}$), the change of micropollutant concentrations (Δ[1,4-dioxane] or Δ[DEET]), and the formation concentration of NDMA (Δ[NDMA]) during the treatments of a solution containing 10 μM Cl-DMA alongside 0.4 μM each of 1,4-dioxane and DEET by 150 μM HOCl and 75 μM NH_4^+ at (a) pH 5.8, (b) pH 7.0, and (c) pH 8.3. The pH of the solutions was maintained by 10 mM phosphates. Error bar represents the range of duplicate experiments.

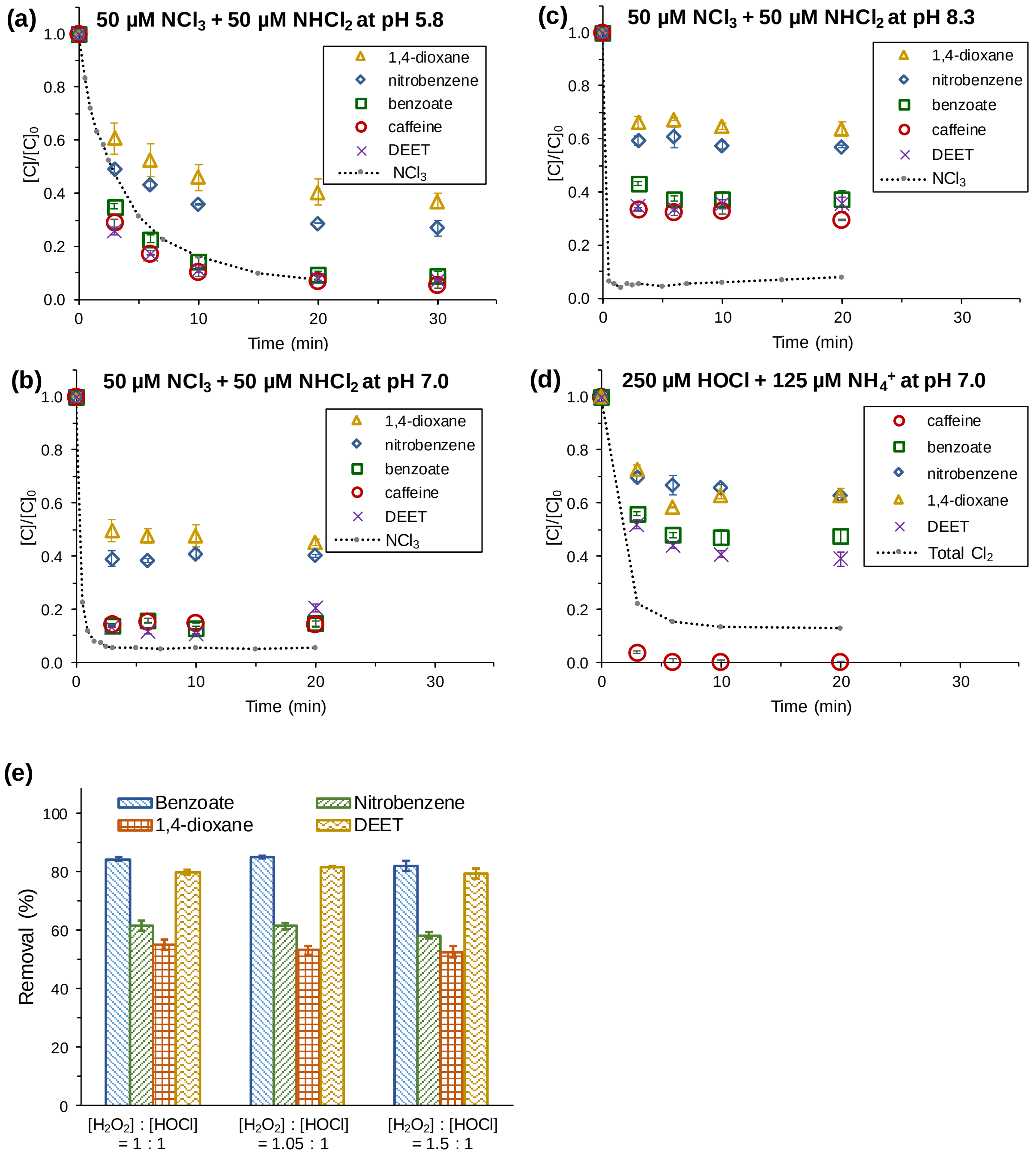

Figure S8. Micropollutant concentrations during the treatment of a mixture of five micropollutants at 0.2 or 0.4 μM each by 50 μM NCl_3 and 50 μM $NHCl_2$ at (a) pH 5.8, (b) pH 7.0, and (c) pH 8.3, and (d) by 250 μM HOCl and 125 μM NH_4^+ at pH 7.0. Error bar represents the range of duplicate experiments. ($[1,4\text{-dioxane}]_0 = [\text{DEET}]_0 = 0.2$ μM, $[\text{benzoate}]_0 = [\text{nitrobenzene}]_0 = [\text{caffeine}]_0 = 0.4$ μM). NCl_3 decomposition were measured in separate experiments without the micropollutants using a UV-spectrum-based approach (see Text S5). Total chlorine concentrations were measured using the DPD method. Also presented in figure (e) is the micropollutant removals during the treatment of a mixture of 1,4-dioxane (0.2 μM), DEET (0.2 μM), benzoate (0.4 μM), and nitrobenzene (0.4 μM) in 20 mM phosphate buffer at pH 7 by 50 μM NCl_3 + 50 μM $NHCl_2$ for 20 min, with different H_2O_2 to HOCl molar ratio to quench the HOCl residual prior to the experiments. The comparable removal of micropollutants under different experimental conditions demonstrated that the presence of a small amount of H_2O_2 did not impact •OH formation during NCl_3-$NHCl_2$ interactions.

Text S4. Determining the reaction rate constant of NCl_3 with micropollutants and evaluating the contribution of NCl_3 in degrading micropollutants during the NCl_3-$NHCl_2$ treatments.

Previous research has reported the high reactivity of NCl_3 with certain compounds, with reported *k* reaching approximately 10^3 $M^{-1}s^{-1}$ for aromatic compounds.[20] To assess to which extent NCl_3 degrades compounds in the NCl_3-$NHCl_2$ experiments, we conducted a preliminary test in which a solution of five micropollutants at 0.2 (1,4-dioxane and DEET) or 0.4 µM (benzoate, nitrobenzene, and caffeine) were treated by 50 µM NCl_3 alone for 20 min. Experiments were conducted in 20 mM phosphate buffer at pH 6.0, in which NCl_3 is stable. Figure S9a shows negligible degradations for 1,4-dioxane and DEET after the treatments, while >15% decompositions were observed for benzoate, nitrobenzene, and caffeine. Further experiments were therefore conducted to experimentally measured the reaction rate constant of NCl_3 (k_{NCl3}) for the three compounds.

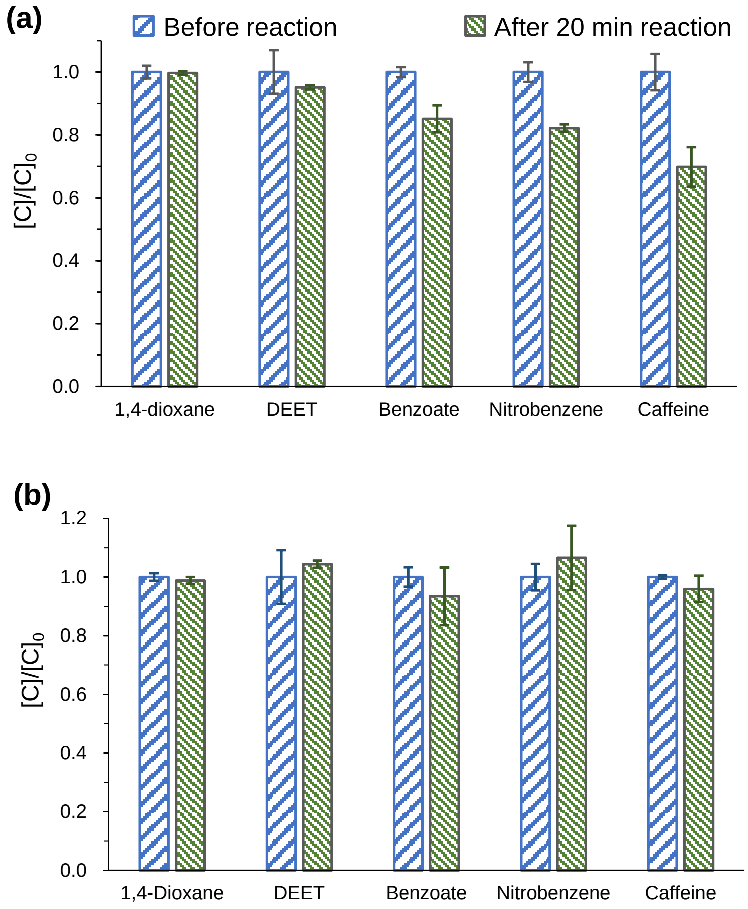

Figure S9. $[C]/[C]_0$ for compounds before and after the treatment of a mixture of five compounds at 0.2 (1,4-dioxane and DEET) or 0.4 µM (benzoate, nitrobenzene, and caffeine) by (a) 50 µM NCl_3 alone or (b) 50 µM $NHCl_2$ alone for 20 min. Error bars represent data range from duplicate experiments. Experiments were conducted in 20 mM phosphate buffer at pH 6.0, in which NCl_3 or $NHCl_2$ is stable.

The reaction rate constants for NCl_3 with micropollutants ($k_{NCl3,\ C}$) were determined by measuring the initial decay rate of micropollutants during the treatments of a solution containing caffeine, nitrobenzene, and benzoate at 1 µM each in 20 mM phosphate buffer at pH 6.0, at which NCl_3 is stable, with excess amount of NCl_3 (i.e., 30, 60, 90, and 120 µM). The initial decay rate of a micropollutant was calculated by Eq. S2. In Eq. S2, C denotes the target micropollutant, and t represents the reaction time. While $[C]_0$ is the initial concentration of micropollutant (which was 1 µM), $[C]_t$ is the

concentration of micropollutant after reaction time t. In these experiments, the reaction time was 3 or 10 min, at which the decay of micropollutant was approximately 10%.

The k_{NCl3} for a micropollutant can be obtained via Eq. S3, in which $[C]_{ini}$ denotes the initial concentration of the micropollutant (1 µM) and $[NCl_3]_{ini}$ represents the initial concentration of NCl_3.

$$\text{initial decay rate} \left(\frac{M}{s}\right) = \frac{\Delta[C]}{\Delta t} = \frac{([C]_0 - [C]_t)}{t} \qquad \text{(Eq. S2)}$$

$$k_{NCl_3,C}\ (M^{-1}s^{-1}) = \frac{\text{initial decay rate for C}}{[C]_{ini} \cdot [NCl_3]_{ini}} \qquad \text{(Eq. S3)}$$

Figure S10 shows the relationships between initial NCl_3 concentration and the initial decay rate of a compound. The initial decay rate of a compound linearly increased as increasing initial NCl_3 concentration. Accordingly, k_{NCl3} values were determined to be 2.1±0.5 $M^{-1}s^{-1}$ for nitrobenzene, 1.9±0.3 $M^{-1}s^{-1}$ for benzoate, and 5.4±0.6 $M^{-1}s^{-1}$ for caffeine.

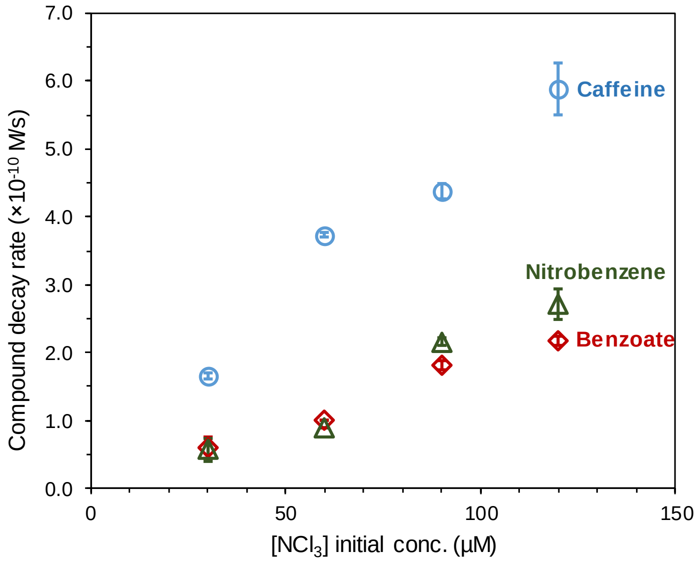

Figure S10. Relationships between initial NCl_3 concentration and the initial decay rate of a compound during the treatments of mixtures of benzoate, nitrobenzene, and DEET at 1 µM each in deionized water buffered (20 mM phosphates) at pH 6.0 by 30–120 µM NCl_3.

The rate constants of NCl_3 with micropollutants were incorporated into the kinetic model developed in this study (Table S6) to evaluate the contribution of NCl_3 to their degradation during the NCl_3-$NHCl_2$ experiments. Model simulation indicated that during the treatment of a mixture of caffeine, nitrobenzene, and benzoate, each at 0.4 µM, by 50 µM NCl_3 + 50 µM $NHCl_2$ at pH 7, the degradations attributable to reactions with NCl_3 were negligible—less than 2 nM for all compounds within 20 minutes, which is less than 0.5% of the initial 0.4-µM concentrations. This is driven by the rapid decomposition of NCl_3 during its reaction with $NHCl_2$ (Figure S11).

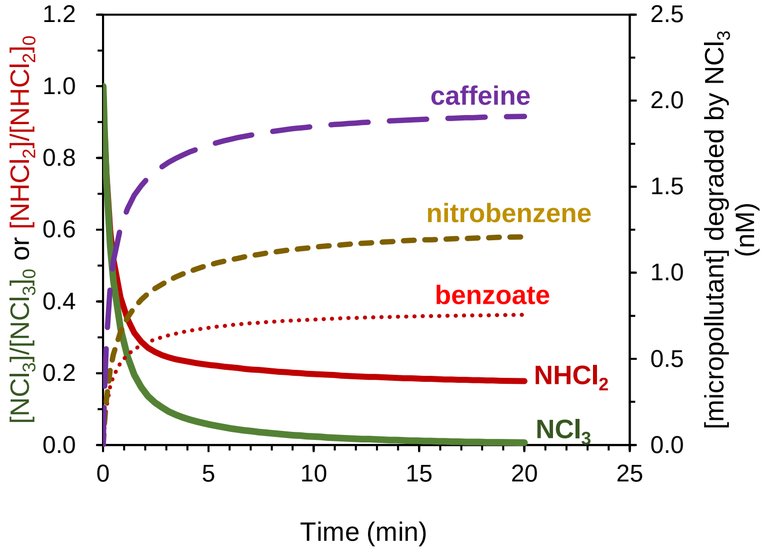

Figure S11. Model simulation of changes in oxidant concentration and micropollutant degradation attributable to reactions with NCl_3 during the treatment of a mixture of caffeine, nitrobenzene, and benzoate (each at 0.4 μM) with 50 μM NCl_3 and 50 μM $NHCl_2$ at pH 7.

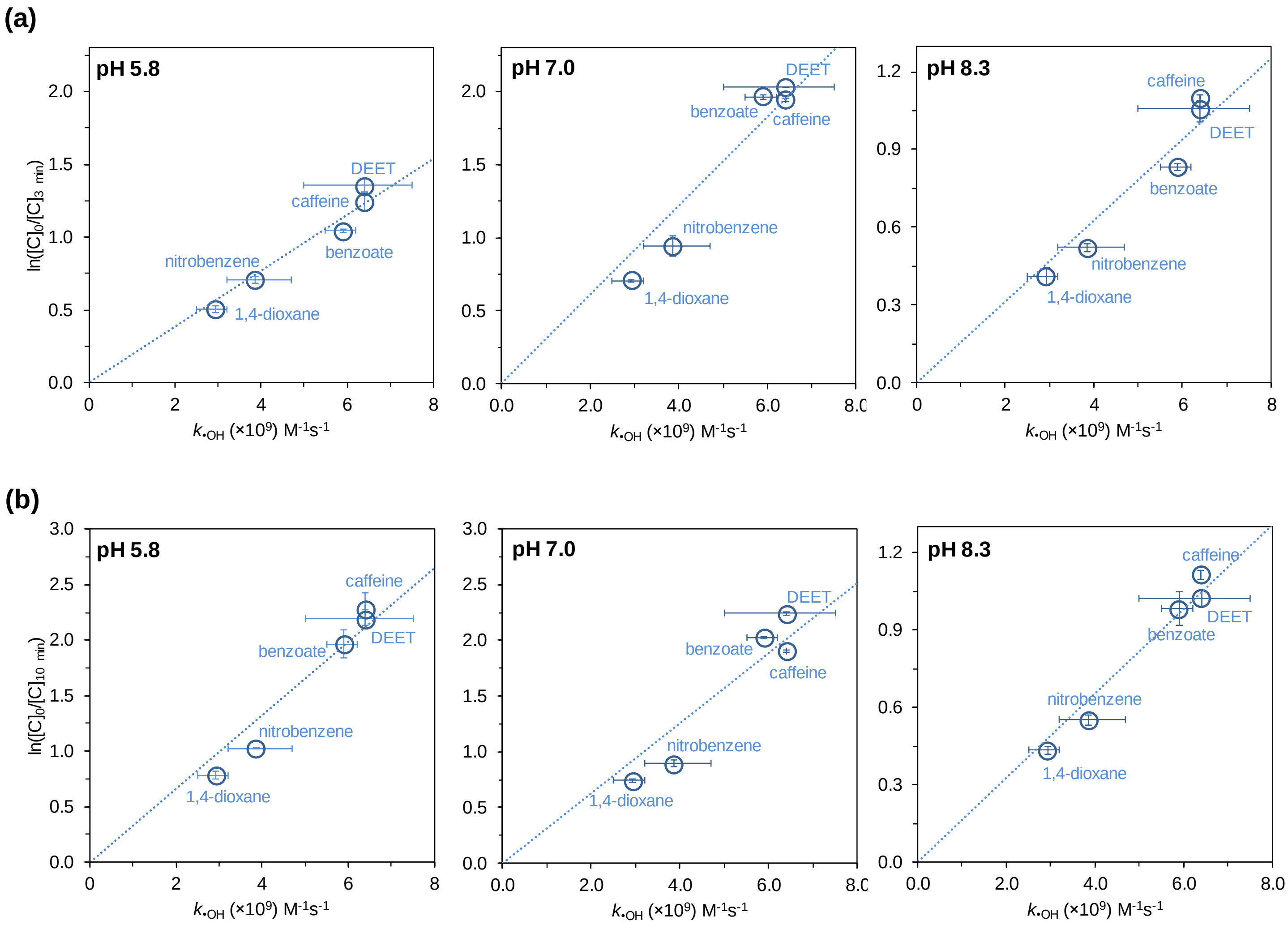

Figure S12. Relationship between $k_{\bullet OH}$ for compounds and (a) the $\ln([C]_0/[C]_{3\ min})$ and (b) the $\ln([C]_0/[C]_{10\ min})$ for compounds in the treatments of the mixture of five micropollutants by 50 μM NCl_3 + 50 μM $NHCl_2$ at pH across 5.8–8.3. The pH of the solutions was maintained by 20 mM phosphates. $[\text{1,4-dioxane}]_0 = [\text{DEET}]_0 = 0.2$ μM, $[\text{benzoate}]_0 = [\text{nitrobenzene}]_0 = [\text{caffeine}]_0 = 0.4$ μM). Dashed lines represent the linear regression for the five compounds.

Text S5. Using a UV-spectrum-based approach to evaluate the evolution and decomposition of oxidants during the reaction of 50 μM NCl_3 with 50 μM $NHCl_2$.

A UV-spectrum/simultaneous equation approach was developed to more accurately quantify the concentrations of chlorine species during the reaction of NCl_3 with $NHCl_2$ or during the breakpoint chlorination in our previous study.[5] Briefly, the reaction of NCl_3 with $NHCl_2$ was conducted in a 10 cm pathlength quartz cuvette mounted in an Agilent Cary 60 spectrophotometer for *in situ* measurements of UV-vis spectra at wavelengths between 250–400 nm. Then, UV spectra were taken periodically during the reaction. For each spectrum, the concentrations of free chlorine (HOCl and OCl^-), NH_2Cl, $NHCl_2$, and NCl_3 were calculated based on the absorbance values at 250, 270, 295, and 360 nm using their corresponding molar absorption coefficients, as presented in Eqs. S4–S8.[5]

$$A_{250\,nm} = \varepsilon_{HOCl,250nm} \times [HOCl] + \varepsilon_{OCl^-,250nm} \times [OCl^-] + \varepsilon_{NH_2Cl,250nm} \times [NH_2Cl] + \varepsilon_{NHCl_2,250nm} \times [NHCl_2] + \varepsilon_{NCl_3,250nm} \times [NCl_3] \quad \text{(Eq. S4)}$$

$$A_{270\,nm} = \varepsilon_{HOCl,270nm} \times [HOCl] + \varepsilon_{OCl^-,270nm} \times [OCl^-] + \varepsilon_{NH_2Cl,270nm} \times [NH_2Cl] + \varepsilon_{NHCl_2,270nm} \times [NHCl_2] + \varepsilon_{NCl_3,270nm} \times [NCl_3] \quad \text{(Eq. S5)}$$

$$A_{295\,nm} = \varepsilon_{HOCl,295nm} \times [HOCl] + \varepsilon_{OCl^-,295nm} \times [OCl^-] + \varepsilon_{NH_2Cl,295nm} \times [NH_2Cl] + \varepsilon_{NHCl_2,295nm} \times [NHCl_2] + \varepsilon_{NCl_3,295nm} \times [NCl_3] \quad \text{(Eq. S6)}$$

$$A_{360\,nm} = \varepsilon_{HOCl,360nm} \times [HOCl] + \varepsilon_{OCl^-,360nm} \times [OCl^-] + \varepsilon_{NH_2Cl,360nm} \times [NH_2Cl] + \varepsilon_{NHCl_2,360nm} \times [NHCl_2] + \varepsilon_{NCl_3,360nm} \times [NCl_3] \quad \text{(Eq. S7)}$$

$$[OCl^-] = [HOCl] \times \frac{10^{-pK_{a,HOCl}}}{10^{-pH}} \quad \text{(Eq. S8)}$$

To evaluate whether unknown products interfere with the quantification of oxidant species, UV spectra for known oxidant species were reconstructed over a wavelength range of 250–400 nm. This was achieved using calculated oxidant concentrations in conjunction with wavelength-dependent molar absorption coefficients (Eq. S9). The reconstructed spectra were then compared to those measured experimentally. Perfect matches were anticipated only if HOCl/OCl^-, NH_2Cl, $NHCl_2$, and NCl_3 were the predominant absorbers within this wavelength range. Further details and illustrative examples are provided in our previous study.[5] Overall, we confirmed that HOCl/OCl^-, NH_2Cl, $NHCl_2$, and NCl_3 were the predominant absorbers within the wavelength range of 250–400 nm during the reaction of NCl_3 with $NHCl_2$ or during the breakpoint chlorination.[5]

$$A_\lambda = \varepsilon_{\lambda,\,HOCl} \times [HOCl] + \varepsilon_{\lambda,\,OCl-} \times [OCl^-] + \varepsilon_{\lambda,\,NH2Cl} \times [NH_2Cl] + \varepsilon_{\lambda,\,NHCl2} \times [NHCl_2] + \varepsilon_{\lambda,\,NCl3} \times [NCl_3],\ (\lambda = 250\text{–}400\ nm) \quad \text{(Eq. S9)}$$

Figure S13 shows the evolution and decomposition of oxidants during the reaction of 50 μM NCl_3 with 50 μM $NHCl_2$ at pH 7 or 8.3. The results indicated that the concentrations of free chlorine (i.e., [HOCl]+[OCl^-]) were always <11 μM throughout the experiments.

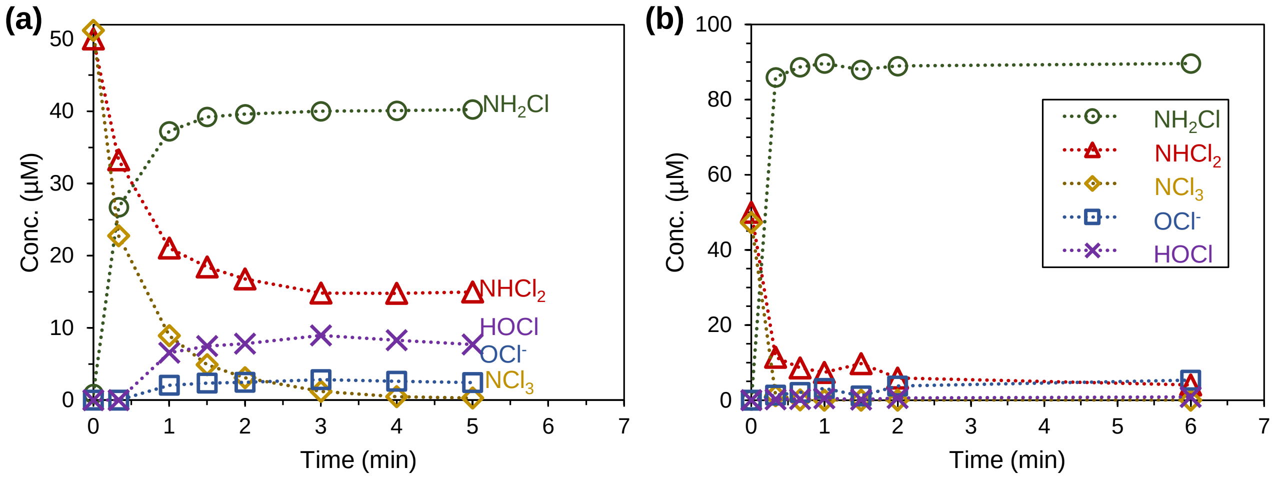

Figure S13. Decomposition and evolution of free chlorine and inorganic chloramines during the reaction of 50 μM NCl_3 with 50 μM $NHCl_2$ at (a) pH 7.0 or (b) pH 8.3 (in 20 mM phosphate buffer). Result for pH 7.0 was adapted from our previous study,[5] while result for pH 8.3 was conducted in this study.

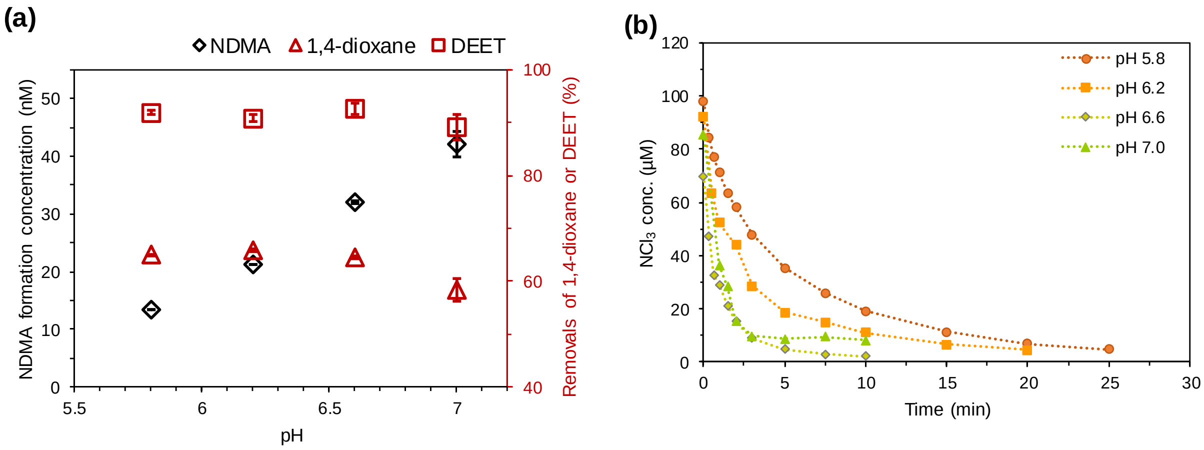

Figure S14. (a) NDMA formation and the removals of 1,4-dioxane or DEET during the treatments of a mixture containing 10 μM Cl-DMA and 0.4 μM each of 1,4-dioxane and DEET at various pH values (buffered using 20 mM phosphates) by 100 μM NCl_3 + 40 μM $NHCl_2$ for 10 or 20 min at which the NCl_3 decomposition was ~90%. (b) NCl_3 concentrations during the reaction of 100 μM NCl_3 with 40 μM $NHCl_2$ at pH 5.8–7.0. The reaction time in sub Fig. a was 20 min for pH 5.8 and 10 min for pH 6.2, 6.6, and 7.0.

Text S6. Experiments assessing whether $O_{2(aq)}$ participates in the reactions for •OH formation during the NCl_3-$NHCl_2$ interaction

We measured the dissolved oxygen (DO) concentrations in deionized water buffered with 20 mM phosphate at pH 7, before and after the treatments with NCl_3 and $NHCl_2$. To initiate the experiment, a 310-mL biological oxygen demand (BOD) bottle was filled with 300 mL of the phosphate buffer. A small aliquot of NCl_3 stock solution was added to achieve the desired concentration, followed by the addition of a stoichiometric amount of H_2O_2 to quench any residual HOCl. The solution was gently mixed using a pipettor. After the addition of $NHCl_2$ at the target concentration, the bottle was immediately sealed, stirred using a magnetic stir bar, and left to stand for 20 minutes to allow for the complete depletion of NCl_3. The cap was then removed after the reaction, and the total chlorine residuals were measured and were quenched by stoichiometric amount of sodium thiosulfate. A DO probe was used to measure the DO concentration. The BOD bottle's narrow mouth minimized contact with ambient air during DO measurement, ensuring accurate readings.

Table S4 summarizes the experimental results. The $O_{2(aq)}$ concentration was 166±1 μM in samples treated with 100 μM NCl_3 and 60 μM $NHCl_2$ for 20 minutes, during which almost all of the 100 μM NCl_3 was consumed. This DO concentration was comparable to that observed in untreated samples (162±1 μM). Notably, comparable DO concentrations were observed in samples before and after treatment with 200 μM NCl_3 and 120 μM $NHCl_2$ for 20 minutes, despite the NCl_3 consumed being 1.2-fold higher than the initial $O_{2(aq)}$ concentration.

Table S4. DO concentration measured before and after the treatments of NCl_3 + $NHCl_2$ in deionized water buffered with 20 mM phosphates at pH 7.0.

Experimental conditions			DO concentration (μM)
$[NCl_3]_{ini}$	$[NHCl_2]_{ini}$	Reaction time (min)	
0	0	-	162±1
100	60	20	166±1
150	90	20	163±7
200	120	20	165±0

Additional experiments were conducted to assess the impact of $O_{2(aq)}$ on micropollutant removals using a mixture of 1,4-dioxane and DEET, each at a concentration of 0.2 μM, in 20 mM phosphate buffer at pH 7. The treatments involved 100 μM NCl_3 and 40 μM $NHCl_2$ for 20 minutes, with and without nitrogen (N_2) purging. In the non-purged samples, the $O_{2(aq)}$ concentration was 268 μM (8.6 mg/L), whereas in the N_2-purged samples, it was 7.2 μM (0.23 mg/L). As shown in Figure S15, the relative differences in micropollutant removals were less than 12% despite the order-of-magnitude difference in $O_{2(aq)}$ concentrations. These findings suggest that $O_{2(aq)}$ does not participate in the reactions of NCl_3 and $NHCl_2$ to generate •OH.

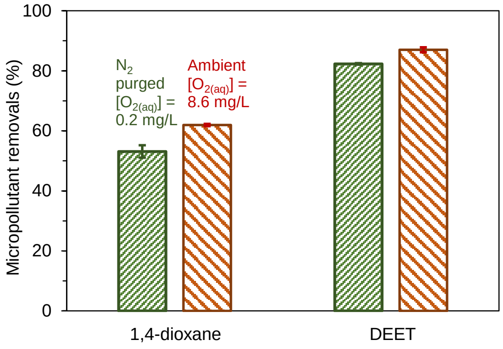

Figure S15. The removals of micropollutants during the treatment of mixtures of 1,4-dioxane and DEET at 0.2 μM each in 20-mM phosphates, with or without N_2 purging, at pH 7 with 100 μM NCl_3 and 40 μM $NHCl_2$ for 20 min.

Text S7. Assessment of the feasibility of using terephthalate as a probe to trace the oxygen source of •OH during the reaction of NCl_3 with $NHCl_2$

Terephthalate is commonly used to quantify •OH formation[21-23] and trace the origin of •OH oxygen (Scheme S1).[11] Initial experiments were conducted to assess whether terephthalate is suitable as a probe compound to trace the oxygen source of •OH during the reaction of NCl_3 with $NHCl_2$. However, we found that 2-hydroxylterephthalate formed during the treatment of terephthalate with NCl_3 alone. For example, Figure S16 shows that treating 9 µM terephthalate in deionized water buffered with 20 mM phosphates at pH 7 by 100 µM NCl_3 alone for 15 min produced 0.58±0.08 µM 2-hydroxylterephthalate, accompanied with the consumption of ~15% of the 9 µM terephthalate. 2-Hydroxylterephthalate formation linearly increased as the parent compound decreased over the 15-min reaction time. This likely occurs via oxidative electron transfer reactions by NCl_3 coupled with hydrolysis (Reaction S1),[22] consistent with previous research indicating NCl_3 can oxidize aromatic compounds with higher rate constants than HOCl.[20] Because NCl_3 coexists with free chlorine and mono-/di-chloramines in breakpoint chlorination, particularly at $Cl_2/N > 2$,[24] our results suggest that •OH quantification may be overestimated when terephthalate is used as the probe compound.

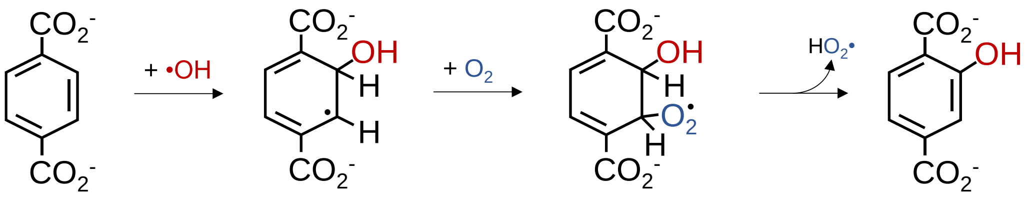

Scheme S1. The reaction mechanisms for the generation of 2-hydroxyterephthalate from the reaction of •OH and terephthalate.[23]

$$R\text{–}C_6H_5 \xrightarrow{-e^-} [R\text{–}C_6H_5]^{\bullet+} \xrightarrow{+OH^-/H_2O} \xrightarrow{-e^-} R\text{–}C_6H_4OH \qquad \text{(Reaction S1)}$$

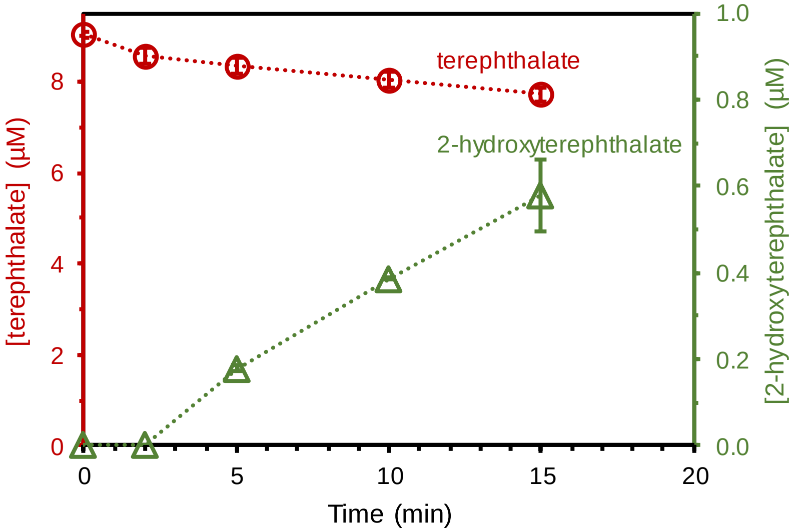

Figure S16. Changes in terephthalate concentrations and formation of 2-hydroxyterephthalate during the treatment of 9 µM terephthalate in 20 mM phosphates at pH 7 by 100 µM NCl_3 alone (with stoichiometric amount of H_2O_2 to quench the HOCl residual first).

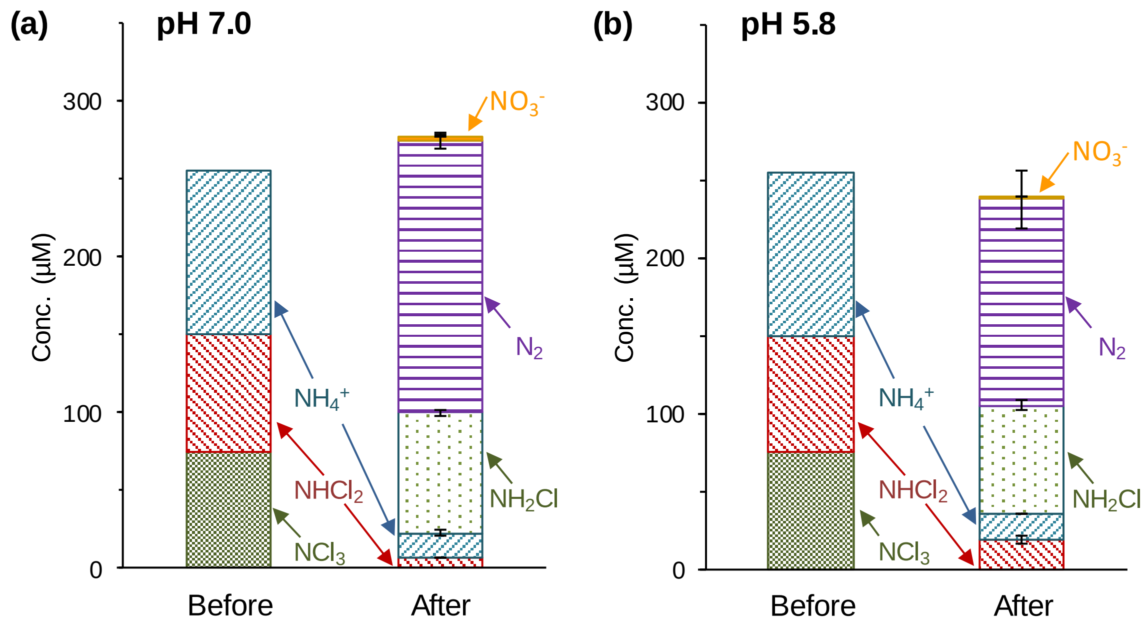

Figure S17. Nitrogen species (N_2, N_2O, NO_3^-, NO_2^-, NH_4^+, NCl_3, $NHCl_2$, NH_2Cl) before and after the treatment of 75 µM ^{15}N-NCl_3 with 75 µM ^{15}N-$NHCl_2$ at (a) pH 7 and (b) pH 5.8 (20 mM phosphate buffered). Stoichiometric amount of H_2O_2 was employed following the addition of NCl_3 to quench the HOCl residual first. Reaction time = 20 min. The addition of 75 µM ^{15}N-$NHCl_2$ would introduce 105 µM ^{15}N-NH_4^+ in the solution, such that the total initial concentration of nitrogen (i.e., $[NCl_3]_{initial}$ + $[NHCl_2]_{initial}$ + $[NH_4^+]_{initial}$) would be 255 µM.

Table S5. Experiments conducted to assess which product (N_2 or NO_3^-) is more closely associated with the generation of •OH (No. 1–14), and experiments conducted to assess the significance of NCl_3-$NHCl_2$ interaction in •OH formation during the breakpoint chlorination reactions (No. 15–18). ^{15}N-NCl_3, ^{15}N-$NHCl_2$, and ^{15}N-NH_4^+ were used in these tests.

Experiment No.	pH	$[^{15}N\text{-}NCl_3]_{initial}$	$[^{15}N\text{-}NHCl_2]_{initial}$	$[Benzene]_{initial}$
1	5.8	100	0	120
2	5.8	100	20	120
3	5.8	100	40	120
4	5.8	100	60	120
5	5.8	100	80	120
6	5.8	100	100	120
7	7.0	100	0	120
8	7.0	100	20	120
9	7.0	100	40	120
10	7.0	100	60	120
11	7.0	100	80	120
12	7.0	100	100	120
13	7.0	0	100	120
14	5.8	0	100	120

Experiment No.	pH	$[HOCl]_{initial}$	$[^{15}N\text{-}NH_4^+]_{initial}$	$[Benzene]_{initial}$
15	7.0	0	0	80
16	7.0	100	50	80
17	7.0	150	75	80
18	7.0	200	100	80

Text S8. Evaluation of the reactivity of benzene toward reactive nitrogen species that potentially exist in breakpoint chlorination reactions or in the reaction of NCl_3 with $NHCl_2$.

Previous research has suggested the formation of reactive nitrogen species (RNS) during breakpoint chlorination reactions, including peroxynitrite/peroxynitrous acid, •NO, and $•NO_2^-$.[3, 4, 25] These reactive species may be generated in the reaction between NCl_3 and $NHCl_2$, in addition to •OH.

To determine whether benzene is a suitable surrogate for the quantification of •OH during the reaction of NCl_3 with $NHCl_2$, we experimentally examined the reactivities of benzene with peroxynitrite/peroxynitrous acid, •NO, and $•NO_2^-$ (collectively RNS). Benzene would be considered a suitable surrogate for the quantification of •OH if it exhibits negligible reactivity toward RNS.

We first prepared a peroxynitrite stock solution following the method described in the literature.[11, 26] This involved mixing 100 mM of H_2O_2 (25 mL) with 100 mM $NaNO_2$ solution (25 mL) in an ice bath. The mixture was allowed to stand in the ice bath until the temperature dropped to approximately 5 °C. Then, 25 mL of 1 M HCl was added to the $H_2O_2/NaNO_2$ mixture, immediately followed by the addition of 25 mL of 1.5 M NaOH; this addition raised the pH of the solution to an alkaline level, halting the reaction. Note that both the HCl and NaOH solutions were kept at 4 °C before use to minimize temperature fluctuations when preparing the peroxynitrite stock solution. The peroxynitrite stock solution was standardized spectrometrically ($\varepsilon_{302\ nm}$ = 1650 $M^{-1}cm^{-1}$).[11, 26] The peroxynitrite concentration was found to be 8.4 mM, equivalent to a 33% yield relative to NO_2^-.

Experiments were conducted by treating a 6 or 12 µM benzene solution, buffered at pH 7.0 (20 mM phosphates), with 100 µM peroxynitrite. While peroxynitrite is stable at alkaline pH (as in the stock solution), it rapidly decomposes at neutral pH (as when spiked into the reaction solution). The decomposition of peroxynitrite generates •OH, •NO, and $•NO_2^-$ through the reactions depicted in Reactions S2–S5. Since the peroxynitrite stock solution contained NO_2^- at a 2:1 NO_2^-: $OONO^-$ molar ratio, the background 200 µM NO_2^- facilitates •OH scavenging, forming $•NO_2$ during the decomposition reactions of peroxynitrite (Reaction S6).[27] The solutions were allowed to stand for 10 min before the analyses of benzene concentrations. Figure S18 shows that benzene concentrations remained unchanged after treatment with peroxynitrite, even with a peroxynitrite concentration far exceeding that of benzene. These results suggest that benzene is not reactive with reactive nitrogen species (RNS) such as HOONO, $OONO^-$, •NO, $•NO_2$. Therefore, benzene can be a suitable surrogate for the quantification of •OH in the reaction of NCl_3 with $NHCl_2$.

$OONO^- + H^+ \rightarrow HOONO$ (pK_a for HOONO = 6.8) (Reaction S2)

$HOONO \rightarrow HNO_3$ (isomerization) (Reaction S3)

$HOONO \rightarrow •OH + •NO_2$ (Reaction S4)

$OONO^- \rightarrow •O_2^- + •NO$ (Reaction S5)

$•OH + NO_2^- \rightarrow + •NO_2 + OH^-$ $k=1.1\times10^{10}\ M^{-1}s^{-1}$ (Reaction S6)

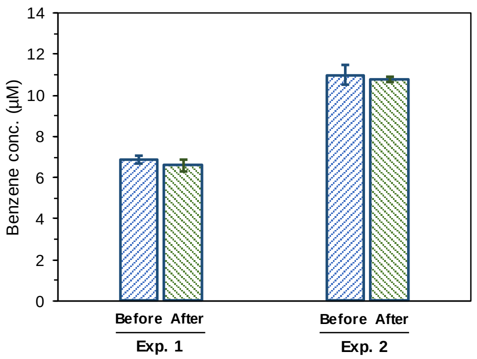

Figure S18. Benzene concentrations in solutions containing 6 µM (Exp. 1) or 12 µM (Exp. 2) benzene at pH 7.0 (buffered with 20 mM phosphate), before and after treatment with 100 µM peroxynitrite. Error bars represent the standard deviation from experimental triplicates (n=3).

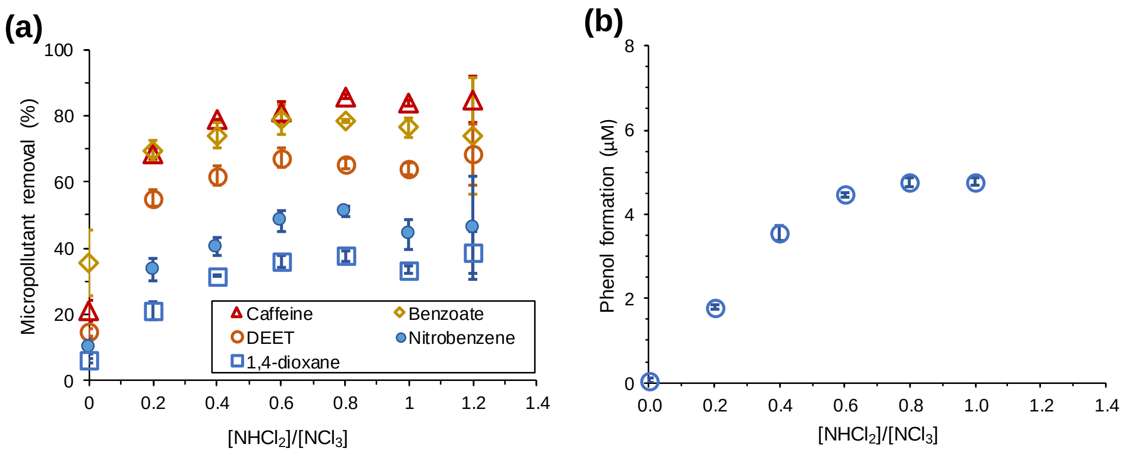

Figure S19. (a) Micropollutant removals during the treatments of a mixture of micropollutants at 0.4 µM each at pH 5.8 (20 mM phosphates) with 50 µM NCl_3 and 0–60 µM $NHCl_2$ (such that the [$NHCl_2$]/[NCl_3] molar ratio ranged from 0 to 1.2) for 20 min. (b) Phenol formation concentrations during the treatment of 100 µM benzene at pH 5.8 (20 mM phosphates) with 100 µM NCl_3 and 0–100 µM $NHCl_2$ (such that the [$NHCl_2$]/[NCl_3] molar ratio ranged from 0 to 1.0) for 20 min.

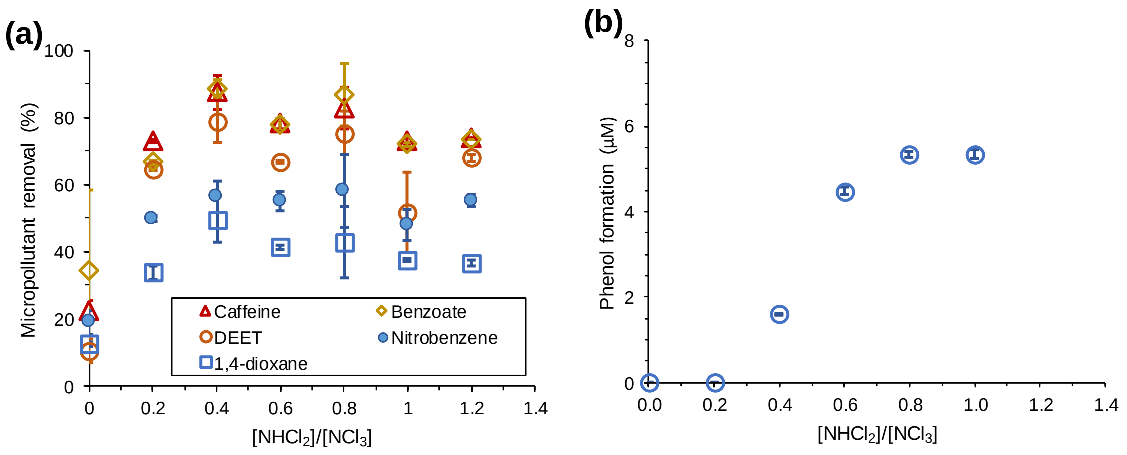

Figure S20. (a) Micropollutant removals during the treatments of a mixture of micropollutants at 0.4 µM each at pH 7.0 (20 mM phosphates) with 50 µM NCl_3 and 0–60 µM $NHCl_2$ (such that the [$NHCl_2$]/[NCl_3] molar ratio ranged from 0 to 1.2) for 20 min. (b) Phenol formation concentrations during the treatment of 100 µM benzene at pH 7.0 (20 mM phosphates) with 100 µM NCl_3 and 0–100 µM $NHCl_2$ (such that the [$NHCl_2$]/[NCl_3] molar ratio ranged from 0 to 1.0) for 20 min.

Text S9. Development and validation of kinetic model.

We recently developed a kinetic model that successfully predicts the evolution and decomposition of oxidants during the reaction of NCl_3 with $NHCl_2$, as well as during breakpoint chlorination.[5] This model is based on the widely used UF model, with modifications including redetermined rate constants for the reactions between NCl_3 and $NHCl_2$, and between NCl_3 and NH_2Cl at pH 7. Additionally, it incorporates an extra branch reaction that elucidates the formation of a nitrosating agent (No. 16 in Table S6).[5] Essentially, the model encompasses the reactions listed as Nos. 1–25 in Table S6, except for Nos. 14 and 15, which were modified based on the results of this study.

Additional elementary reactions involving the formation and decomposition of phenol were integrated into the kinetic model. These reactions include the reactions for phenol formation and decomposition with oxidants relevant to breakpoint chlorination, as discussed in the main text. These reactions are listed as Nos. 26–35.

Formation of phenol from the reaction of •OH with benzene involves multiple steps. •OH adds to benzene to form a hydroxycyclohexadienyl radical (C_6H_6OH•) (No. 26 in Table S6) with a near-diffusion-controlled rate constant (7.9×10^9 $M^{-1}s^{-1}$). C_6H_6OH• undergoes reversible O_2 addition to generate hydroxycyclohexadienylperoxyl radicals (C_6H_6OOOH•; k=3.9×10^8 $M^{-1}s^{-1}$) (No. 27 in Table S6). C_6H_6OOOH• decomposes to phenol and HO_2• (No. 28 in Table S6), among other products (No. 29 in Table S6). Although the rate constant for C_6H_6OOOH• decomposition is not available, a previous study reported a 45(±3)% yield of phenol with respect to the benzene consumed.[28] Therefore, we have added reaction Nos. 28 and 29 in the kinetic model. Assuming the reaction Nos. 28 and 29 are not the bottleneck reactions, we assigned a rate constant of 4.50×10^9 s^{-1} for reaction No. 28 and of 5.50×10^9 s^{-1} for reaction No. 29. In this case, the yield of phenol relative to benzene loss would be 45%, as reported in the literature.[28] Lastly, •OH scavenging by oxidants (listed as Nos. 36–40 in Table S6) may impact the removal efficiencies of micropollutants. Those reactions were also incorporated into the model.

The UF model developed by Jafvert and Valentine adapted the pathway and reaction rate constant for the reaction of NCl_3 with $NHCl_2$ from Hand and Margerum's work[29] and Yiin and Margerum's work.[30] Formation of HOCl and N_2 from the reaction of NCl_3 and $NHCl_2$ involve multiple steps, including the formation and decomposition and intermediates, and H_2O/OH^- participate in HOCl formation. The reaction scheme is similar to Scheme 1 presented in the main text. This reaction scheme assumes that the initial reaction between NCl_3 and $NHCl_2$ to form the intermediate is the rate-limiting step, and an unbalanced reaction U12 (or No. 14 in Table S6; $NCl_3 + NHCl_2 \rightarrow 2\ HOCl + N_2$) was incorporated in the UF model. While the rate constant for this reaction (k for No. 14) was reported to be 5.6×10^3 $M^{-1}s^{-1}$ at pH 7, our previous study found that this rate constant significantly overestimated the decay of NCl_3 in the reaction of NCl_3 with NH_2Cl. Therefore, our previous study conducted experiments to re-determine the rate constants for the reaction between NCl_3 and $NHCl_2$.

In the kinetic model we developed previously,[5] the rate constant for reaction No. 14 ($NCl_3 + NHCl_2 \rightarrow 2\ HOCl + N_2$) was 530 $M^{-1}s^{-1}$. Since branch reactions exist in the reactions between NCl_3 and $NHCl_2$ through which N_2 forms as an ultimate product, we revised this reaction ($NCl_3 + NHCl_2 \rightarrow 2\ HOCl + N_2$) to $NCl_3 + NHCl_2 \rightarrow 2\ HOCl + N_2 + 0.52$ •OH. To do this, we added additional reaction, reaction No. 15 ($NCl_3 + NHCl_2 \rightarrow 2\ HOCl + N_2 +$ •OH), in the model, and the rate constant for reaction No. 14 to that of reaction No. 15 was set to be 0.48/0.52; k for reaction No. 14 = 253 $M^{-1}s^{-1}$ and k for reaction No. 15 = 277 $M^{-1}s^{-1}$.

$NCl_3 + NHCl_2 \rightarrow 2\ HOCl + N_2$ $\qquad k_{No.14} = 253\ M^{-1}s^{-1}$

$NCl_3 + NHCl_2 \rightarrow 2\ HOCl + N_2 + \bullet OH$ $\qquad k_{No.15} = 277\ M^{-1}s^{-1}$

The kinetics for the formation of HOCl, N_2, and •OH and from reactions No. 14 and No. 15 combined can be expressed by Eqs. S10–S12:

$$\frac{d[HOCl]}{dt} = 2 \times k_{No.14}[NCl_3][NH_2Cl] + 2 \times k_{No.15}[NCl_3][NH_2Cl] \qquad \text{(Eq. S10)}$$

$$\frac{d[N_2]}{dt} = k_{No.14}[NCl_3][NH_2Cl] + k_{No.15}[NCl_3][NH_2Cl] \qquad \text{(Eq. S11)}$$

$$\frac{d[\bullet OH]}{dt} = k_{No.15}[NCl_3][NH_2Cl] \qquad \text{(Eq. S12)}$$

In this case, the molar ratio of the products formed during the reaction of NCl_3 with $NHCl_2$ would be $\frac{d[HOCl]}{dt}$: $\frac{d[N_2]}{dt}$: $\frac{d[\bullet OH]}{dt}$ = 2: 1: 0.52. The stoichiometry of the revised reaction reflects the observation that 1.92 mole formation per mole of benzene degraded (Figure 4 in the main text).

Table S6. Principal reactions in the kinetic model

No.	*k*	Unit for *k*	Reaction	Note	Ref.
Breakpoint chlorination reactions					
1	4.17×10^{6}	$M^{-1}s^{-1}$	$HOCl + NH_3 \rightarrow NH_2Cl + H_2O$	Reaction U1 in Table S1	[6]
2	2.5×10^{-5}	s^{-1}	$NH_2Cl + H_2O \rightarrow HOCl + NH_3$	Reaction U2 in Table S1	[6]
3	2.78×10^{2}	$M^{-1}s^{-1}$	$NH_2Cl + HOCl \rightarrow NHCl_2 + H_2O$	Reaction U3 in Table S1	[6]
4	6.39×10^{-7}	s^{-1}	$NHCl_2 + H_2O \rightarrow NH_2Cl + HOCl$	Reaction U4 in Table S1	[6]
5	2.97×10^{-3}	$M^{-1}s^{-1}$	$NH_2Cl + NH_2Cl \rightarrow NHCl_2 + NH_3$	Reaction U5 in Table S1[a]	[6]
6	6.00×10^{-3}	$M^{-1}s^{-1}$	$NHCl_2 + NH_3 \rightarrow 2\ NH_2Cl$	Reaction U6 in Table S1[a]	[6]
7	2.00×10^{-7}	s^{-1}	$NHCl_2 + H_2O \rightarrow HNO$ + product	Reaction U7 in Table S1[a]	[6]
8	2.78×10^{4}	$M^{-1}s^{-1}$	$NHCl_2 + HNO \rightarrow HOCl$ + product	Reaction U8 in Table S1	[6]
9	8.30×10^{3}	$M^{-1}s^{-1}$	$NH_2Cl + HNO \rightarrow$ product	Reaction U9 in Table S1	[6]
10	1.53×10^{-2}	$M^{-1}s^{-1}$	$NH_2Cl + NHCl_2 \rightarrow$ product	Reaction U10 in Table S1	[6]
11	3.30×10^{9}	$M^{-2}s^{-1}$	$HOCl + NHCl_2 + OH^- \rightarrow NCl_3 + OH- + H_2O$	Reaction U11 in Table S1	[6]
12	1.00×10^{5}	$M^{-2}s^{-1}$	$HOCl + NHCl_2 + OCl^- \rightarrow NCl_3 + OH^- + HOCl$	Reaction U11 in Table S1	[6]
13	1.60×10^{4}	$M^{-2}s^{-1}$	$HOCl + NHCl_2 + HPO_4^{2-} \rightarrow NCl_3 + OH^- + H_2PO_4^-$	Reaction U11 in Table S1	[6]
14	2.52×10^{2}	$M^{-1}s^{-1}$	$NCl_3 + NHCl_2 \rightarrow 2\ HOCl + N_2$	Revised reaction U12 in Table S1[a]	[5]
15	2.77×10^{2}	$M^{-1}s^{-1}$	$NCl_3 + NHCl_2 \rightarrow 2\ HOCl + N_2 + \bullet OH$	Revised reaction U12 in Table S1[a]	[5]
16	2.54×10^{2}	$M^{-1}s^{-1}$	$NCl_3 + NHCl_2 \rightarrow NHCl_2 + ClNO + H^+ + Cl^-$	Revised reaction U12 in Table S1[a]	[5]
17	3.10×10^{0}	$M^{-1}s^{-1}$	$NCl_3 + NH_2Cl \rightarrow HOCl$ + product	Reaction U13 in Table S1[a]	[5]
18	1.00×10^{-3}	s^{-1}	$H_2O \rightarrow H^+ + OH^-$	pK_a for H_2O at 25 °C = 14	[5]
19	1.00×10^{11}	$M^{-1}s^{-1}$	$H^+ + OH^- \rightarrow H_2O$	pK_a for H_2O at 25 °C = 14	[5]
20	1.41×10^{3}	s^{-1}	$HOCl \rightarrow OCl^- + H^+$	pK_a for HOCl at 25 °C = 7.5	[5]
21	5.00×10^{10}	$M^{-1}s^{-1}$	$OCl^- + H^+ \rightarrow HOCl$	pK_a for HOCl at 25 °C = 7.5	[5]
22	5.00×10^{10}	$M^{-1}s^{-1}$	$H^+ + Cl^- \rightarrow HCl$	pK_a for HCl at 25 °C = -6.2	[5]
23	8.60×10^{16}	s^{-1}	$HCl \rightarrow H^+ + Cl^-$	pK_a for HCl at 25 °C = -6.2	[5]
24	2.51×10^{1}	s^{-1}	$NH_4^+ \rightarrow NH_3 + H^+$	pK_a for NH_4^+ at 25 °C = 9.3	[5]
25	5.00×10^{10}	$M^{-1}s^{-1}$	$NH_3 + H^+ \rightarrow NH_4^+$	pK_a for NH_4^+ at 25 °C = 9.3	[5]
Phenol/phenolate formation reactions					
26	7.80×10^{9}	$M^{-1}s^{-1}$	$\bullet OH + C_6H_6 \rightarrow C_6H_6OH\bullet$	Reaction R10 in the main text	[8]
27	4.00×10^{8}	$M^{-1}s^{-1}$	$C_6H_6OH\bullet + O_2 \rightarrow C_6H_6OOOH\bullet$	Reaction R11 in the main text	[31]
28	4.50×10^{9}	s^{-1}	$C_6H_6OOOH\bullet \rightarrow C_6H_5OH$ + product	Reaction R12 in the main text	this study
29	5.50×10^{9}	s^{-1}	$C_6H_6OOOH\bullet \rightarrow$ product	Reaction R13 in the main text	this study
30	1.12×10^{0}	s^{-1}	$C_6H_5OH \rightarrow H^+ + C_6H_5O^-$	Reaction R14 in the main text	assumed[b]
31	1.00×10^{10}	$M^{-1}s^{-1}$	$H^+ + C_6H_5O^- \rightarrow C_6H_5OH$	Reaction R14 in the main text	assumed[b]
32	5.00×10^{-1}	$M^{-1}s^{-1}$	$C_6H_5OH + HOCl \rightarrow$ product		[32]
33	3.52×10^{4}	$M^{-1}s^{-1}$	$C_6H_5O^- + HOCl \rightarrow$ product	Reaction R16 in the main text	[32]
34	5.00×10^{-2}	$M^{-1}s^{-1}$	$C_6H_5OH + NCl_3 \rightarrow$ product		[20]
35	3.89×10^{5}	$M^{-1}s^{-1}$	$C_6H_5O^- + NCl_3 \rightarrow$ product	Reaction R15 in the main text	[20]
Radical scavenging reactions					
36	1.00×10^{10}	$M^{-1}s^{-1}$	$\bullet OH + C_6H_5OH \rightarrow$ product		[13]
37	1.21×10^{9}	$M^{-1}s^{-1}$	$\bullet OH + HOCl \rightarrow$ product		[33]
38	6.37×10^{9}	$M^{-1}s^{-1}$	$\bullet OH + OCl- \rightarrow$ product		[33]
39	1.02×10^{9}	$M^{-1}s^{-1}$	$\bullet OH + NH_2Cl \rightarrow$ product		[34]
40	6.21×10^{8}	$M^{-1}s^{-1}$	$\bullet OH + NHCl_2 \rightarrow$ product		[35]

[a] rate constant at pH 7; [b] pK_a for phenol = 9.99 at 25 °C

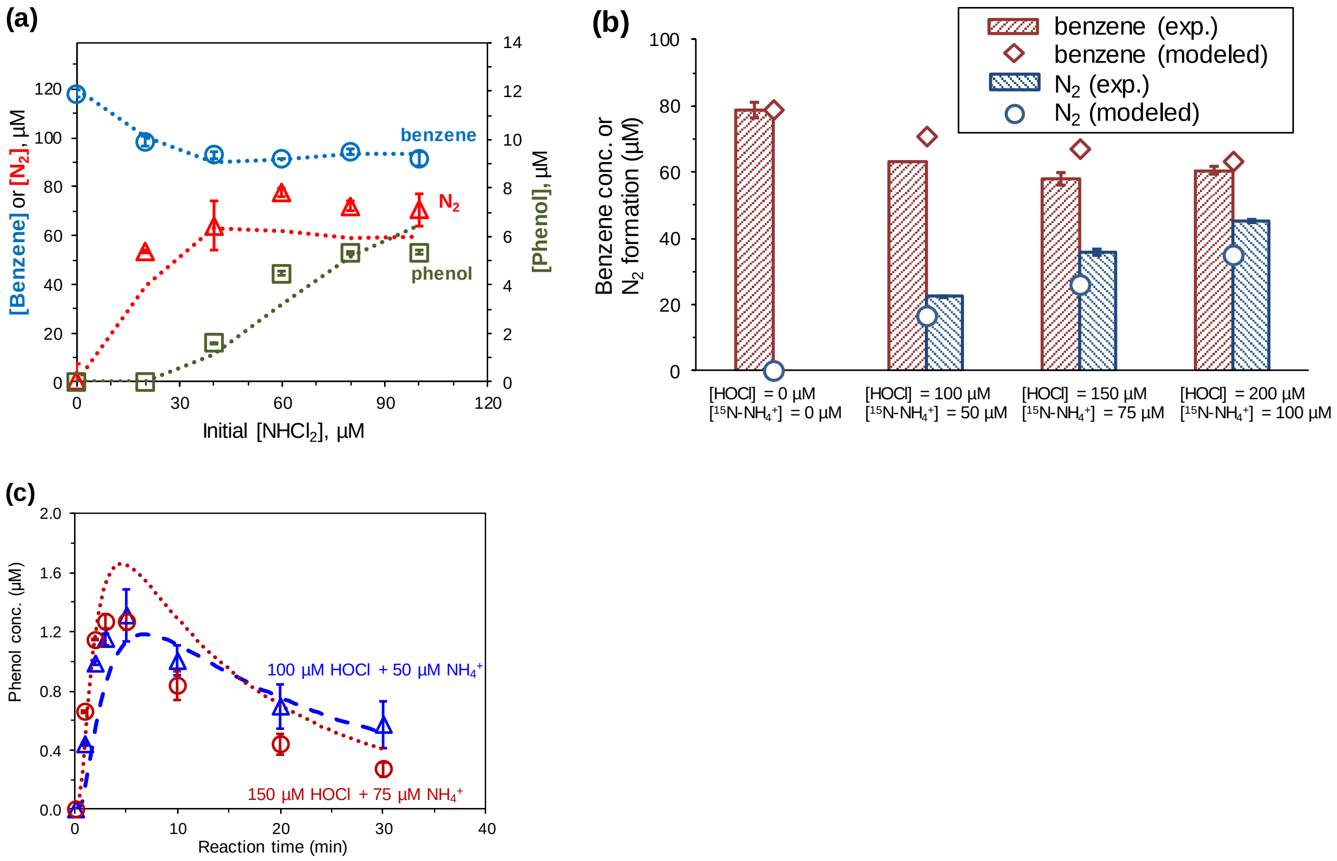

Figure S21. (a) Modeled and measured concentrations of benzene, N_2 (^{15}N-N_2), and phenol during the treatment of a mixture of 120 µM benzene with 100 µM ^{15}N-NCl_3 and 0–100 µM ^{15}N-$NHCl_2$ for 20 minutes. (b) Modeled and measured concentrations of benzene and N_2 (^{15}N-N_2) during the treatment of a mixture of 80 µM benzene with 0–200 µM HOCl and 0–100 µM ^{15}N-NH_4^+ for 30 minutes. (c) Modeled and measured concentrations of phenol during the treatment of a mixture of 80 µM benzene with 100 µM HOCl and 50 µM ^{14}N-NH_4^+ or with 150 µM HOCl and 75 µM ^{14}N-NH_4^+ for 30 minutes. Error bar represents the range from experimental duplicates.

Text S10. Using *tert*-Butanol as a probe to detect •OH formation during the reaction of NCl_3 with $NHCl_2$.

The second probe we employed alongside benzene to assess •OH formation is tert-butanol ($(CH_3)_3COH$). When reacting with •OH, *tert*-butanol primarily produces formaldehyde, with a well-established yield of 30±4%.[36] •OH reacts with *tert*-butanol mainly by H-abstracting from carbon (95%) and, to a lesser extent, from oxygen (5%).[37] Abstraction from carbon forms an alkyl radical (•$CH_2C(CH_3)_2OH$) (Reaction S7), which, in the presence of dissolved oxygen ($O_{2(aq)}$), reacts with O_2 to form a peroxyl radical (•$OOCH_2C(CH_3)_2OH$) (Reaction S8). This radical undergoes self-recombination, creating a short-lived tetroxide intermediate ($[OOCH_2C(CH_3)2OH]_2$) (Reaction S9). The decomposition of this intermediate generates formaldehyde (Reaction S10), among other products (Reaction S11).[36]

Unlike phenol (the main product of benzene's reaction with •OH), formaldehyde quickly hydrolyzes to methanediol ($CH_2(OH)_2$), which has low reactivity with oxidants (e.g., the reaction rate constant k_{O3} for $CH_2(OH)_2$ is only 0.1 $M^{-1}s^{-1}$).[38] This suggests that methanediol produced from the reaction of *tert*-butanol with •OH is unlikely to be degraded by oxidants and can therefore be reliably used to quantify •OH formation during the reaction of NCl_3 with $NHCl_2$. Our control experiments confirmed that methanediol exhibits low reactivity with NCl_3, $NHCl_2$, and H_2O_2. Since the reaction rate constants and formaldehyde yields (30±4%) were well-established in the literature, formation of formaldehyde can be predicted using the kinetic model with these key reactions incorporated.

$CH_3)_3COH$ + •OH → •$CH_2C(CH_3)_2OH$	$k = 6.6\times10^8\ M^{-1}s^{-1}$	(Reaction S7)[13]
•$CH_2C(CH_3)_2OH + O_2$ → •$OOCH_2C(CH_3)_2OH$	$k = 2\times10^9\ M^{-1}s^{-1}$	(Reaction S8)[36]
•$OOCH_2C(CH_3)_2OH$ + •$OOCH_2C(CH_3)_2OH$ → $[OOCH_2C(CH_3)_2OH]_2$	$k = 4\pm1\times10^8\ M^{-1}s^{-1}$	(Reaction S9)[39]
$[OOCH_2C(CH_3)_2OH]_2 \rightarrow O_2 + 2\ HCHO + 2$ •$C(CH_3)_2OH$	$k = 3(\pm0.4)\times10^5\ s^{-1}$	(Reaction S10)[40]
$[OOCH_2C(CH_3)_2OH]_2$ → products	$k = 7\times10^5\ s^{-1}$	(Reaction S11)[40]

Experiments were conducted by treating a 500 µM *tert*-butanol solution in 20 mM phosphate buffer at pH 7.0 by 100 µM NCl_3 with 0–100 µM $NHCl_2$ for 20 min. Stoichiometric amount of thiosulfate quencher was applied to terminate the reaction prior to the analyses of formaldehyde. Note that our control experiments showed that the 500 µM *tert*-butanol did not impact the stability of NCl_3 or $NHCl_2$ over the timescale of 20 min, and the reaction between *tert*-butanol with NCl_3, $NHCl_2$, or H_2O_2 did not produce formaldehyde.

Figure S22 shows that with NCl_3 fixed at 100 µM, increasing $NHCl_2$ concentration from 0 to 40 µM led to a sharp rise in formaldehyde formation, reaching 8.4±0.2 µM. Beyond 40 µM $NHCl_2$, further increases up to 100 µM caused formaldehyde formation to plateau. This trend is consistent with micropollutant removals observed in the NCl_3-$NHCl_2$ experiments (Figures S19-S20).

The kinetic model developed in this study (Text S9) incorporating additional elementary reactions for formaldehyde formation (Reactions S7-S11) accurately predicted formaldehyde concentrations across all experiments (Figure S22). For example, the model predicted a formaldehyde concentration of 10±1.3 µM for 100 µM NCl_3 + 60 µM $NHCl_2$, closely matching the measured concentration of 9.6±0.1 µM. The strong agreement between model predicted values and experimental data further supports that the NCl_3-$NHCl_2$ interactions result in •OH formation.

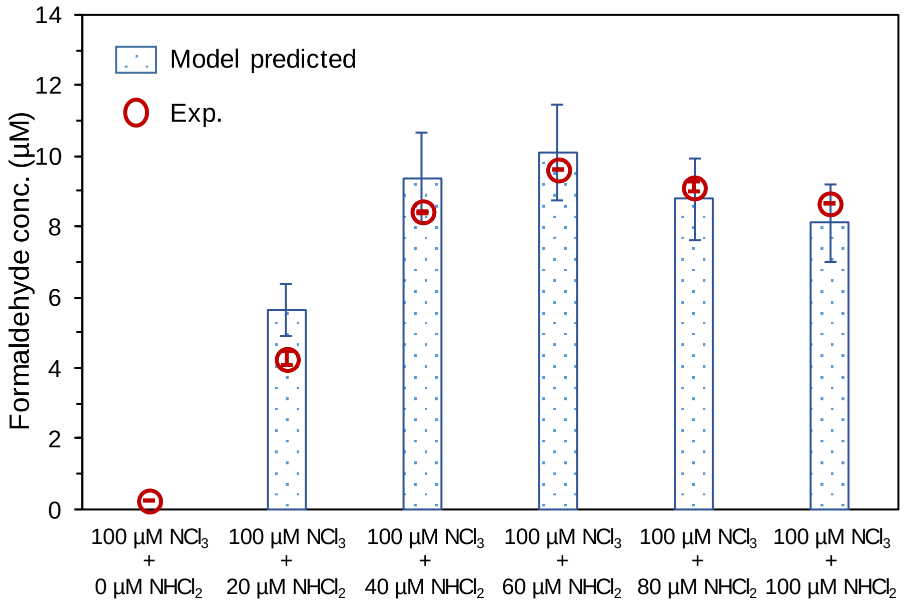

Figure S22. Experimentally measured formaldehyde concentrations versus model-predicted values during the treatment of a 500 µM *tert*-butanol solution, subjected to either breakpoint chlorination (30 min) or NCl_3 + $NHCl_2$ (20 min). Error bars for the model predicted concentrations reflect the range of modeled formaldehyde concentration using the yield of 0.26, 0.3 and, 0.34 (i.e., 30±4%), while error bars for the experimentally measured values represent the data range from duplicate experiments.

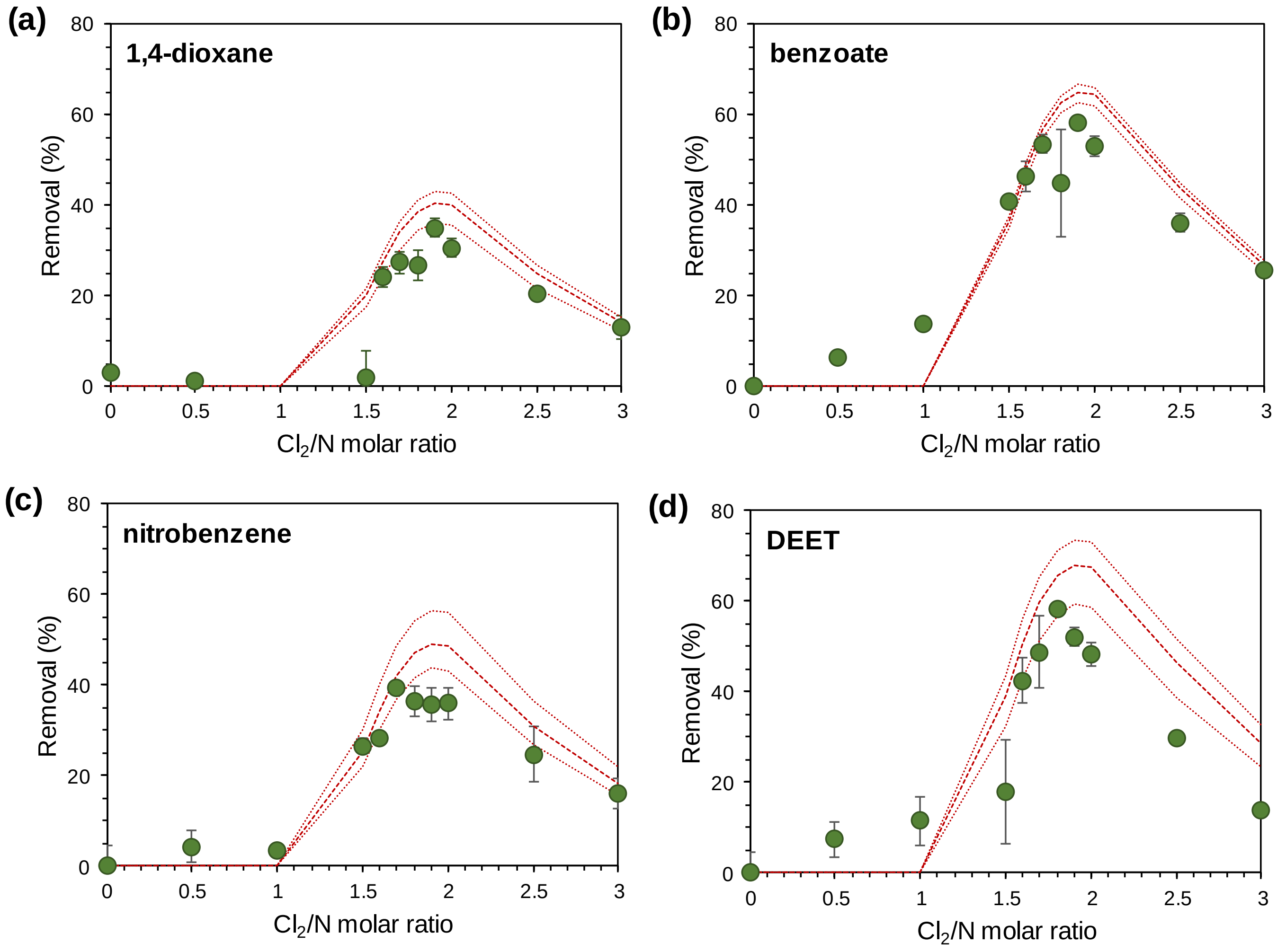

Figure S23. Comparison between the modeled (dash lines) and experimentally measured removals of micropollutants (symbols) during the treatments of a mixture of 0.2 or 0.4 μM micropollutants and 50 μM NH_4^+ by various concentration of HOCl (0–150 μM) at pH 7.0 for 20 min. The pH of the solutions was maintained by 10 mM phosphates. ([1,4-dioxane]$_0$ = [DEET]$_0$ = 0.2 μM, [benzoate]$_0$ = [nitrobenzene]$_0$ = [caffeine]$_0$ = 0.4 μM). Error bar represents the range of duplicate experiments. Red lines are the model predicted removals for micropollutants using the largest (upper line), averaged (middle line), and lowest (bottom line) $k_{\bullet OH}$ values listed in Table S3.

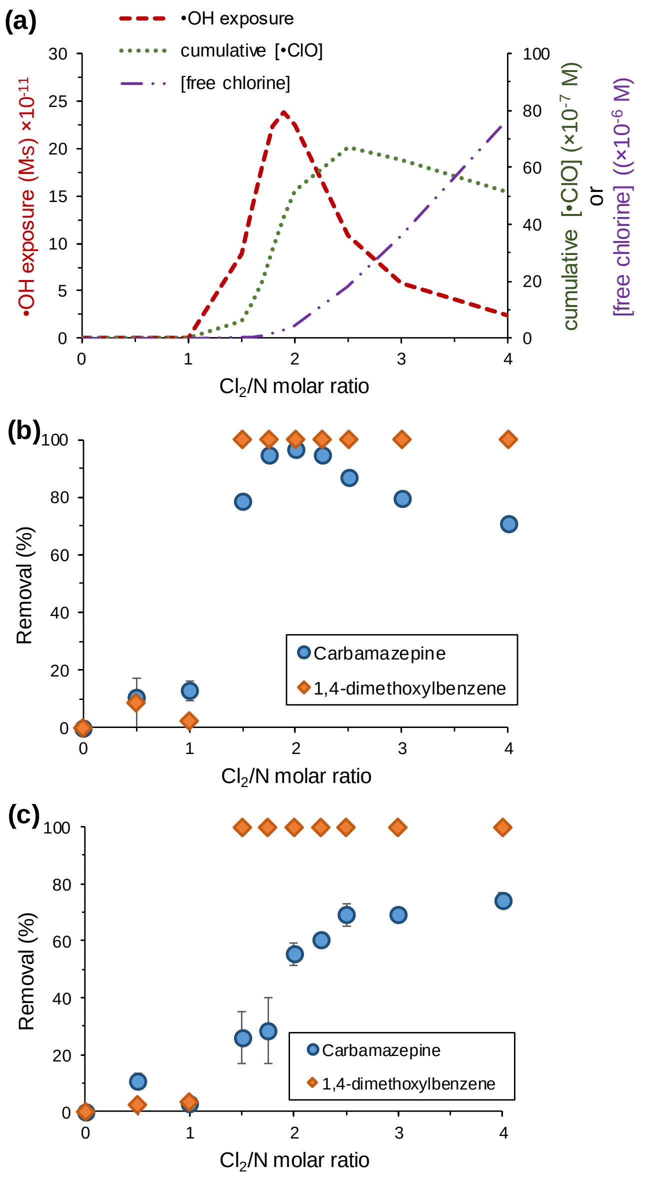

Figure S24. (a) Model simulated •OH exposure, cumulative •ClO formation concentration, and free chlorine concentration at 30 minutes after treating a mixture of 50 µM NH_4^+ in 10 mM phosphate buffer at pH 7 by 0–200 µM HOCl. (b) Experimentally measured micropollutant removals at 30 minutes after treating a mixture of carbamazepine and 1,4-dimethoxylbenzene at 0.5 µM each and 50 µM NH_4^+ in 10 mM phosphate buffer at pH 7 by 0–200 µM HOCl. (c) Experimentally measured micropollutant removals at 30 minutes after treating an authentic drinking water spiked with carbamazepine and 1,4-dimethoxylbenzene at 0.5 µM each and 50 µM NH_4^+ by 0–200 µM HOCl. Basic water qualities for the authentic drinking water are 0.9 mg-C/L for dissolved organic carbon (DOC), 20.6 mg-C/L for dissolved inorganic carbon (DIC), 2.4 µM for NH_4^+, <1 µM for NO_2^-, <1 µM for NO_3^-, and pH was 8.0. Chlorine residual for the authentic drinking water was <0.2 mg/L as Cl_2. Note that an additional 50 µM NH_4^+ was spiked into the authentic drinking water.

References

1. Ye, B.; Wu, Q.-Y.; Wang, W.-L.; Hu, H.-Y., PPCP degradation by ammonia/chlorine: Efficiency, radical species, and byproducts formation. *Water Res.* **2023,** *235*, 119862.
2. Patton, S. D.; Dodd, M. C.; Liu, H., Degradation of 1,4-dioxane by reactive species generated during breakpoint chlorination: Proposed mechanisms and implications for water treatment and reuse. *Journal of Hazardous Materials Letters* **2022,** *3*, 100054.
3. Schreiber, I. M.; Mitch, W. A., Enhanced nitrogenous disinfection byproduct formation near the breakpoint: Implications for nitrification control. *Environ. Sci. Technol.* **2007,** *41*, (20), 7039-7046.
4. Wang, W. L.; Wu, Q. Y.; Du, Y.; Huang, N.; Hu, H. Y., Elimination of chlorine-refractory carbamazepine by breakpoint chlorination: Reactive species and oxidation byproducts. *Water Res.* **2018,** *129*, 115-122.
5. Chuang, Y. H.; Chen, T. Y.; Chou, C. S.; Chu, L. K.; Hou, C. Y.; Szczuka, A., Critical Role of Trichloramine Interaction with Dichloramine for N-Nitrosamine Formation during Breakpoint Chlorination. *Environ. Sci. Technol.* **2023,** *57*, (40), 15232-15242.
6. Jafvert, C. T.; Valentine, R. L., Reaction scheme for the chlorination of ammoniacal water. *Environ. Sci. Technol.* **1992,** *26*, (3), 577-586.
7. Patton, S.; Li, W.; Couch, K. D.; Mezyk, S. P.; Ishida, K. P.; Liu, H., Impact of the ultraviolet photolysis of monochloramine on 1,4-dioxane removal: New insights into potable water reuse. *Environ Sci Technol Lett* **2017,** *4*, (1), 26-30.
8. Buxton, G. V.; Greenstock, C. L.; Helman, W. P.; Ross, A. B., Critical Review of rate constants for reactions of hydrated electrons, hydrogen atoms and hydroxyl radicals ($•OH/•O^-$) in aqueous solution. *J Phys Chem Ref Data* **1988,** *17*, (2), 513-886.
9. Thomas, J., Rates of reaction of the hydroxyl radical. *T Faraday Soc* **1965,** *61*, 702-707.
10. Lei, Y.; Yu, Y.; Lei, X.; Liang, X.; Cheng, S.; Ouyang, G.; Yang, X., Assessing the Use of Probes and Quenchers for Understanding the Reactive Species in Advanced Oxidation Processes. *Environ. Sci. Technol.* **2023,** *57*, (13), 5433-5444.
11. Lu, S.; Shang, C.; Sun, B.; Xiang, Y., Dominant Dissolved Oxygen-Independent Pathway to Form Hydroxyl Radicals and the Generation of Reactive Chlorine and Nitrogen Species in Breakpoint Chlorination. *Environ. Sci. Technol.* **2023,** *57*, (1), 150-159.
12. Lei, Y.; Cheng, S. S.; Luo, N.; Yang, X.; An, T. C., Rate Constants and Mechanisms of the Reactions of $•Cl^-$ and $•Cl_2^-$ with Trace Organic Contaminants. *Environ. Sci. Technol.* **2019,** *53*, (19), 11170-11182.
13. The NIST database. NDRL/NIST Solution Kinetics Database (Web pages; https://kinetics.nist.gov/solution/, 2021-11-12 accessed). http://kinetics.nist.gov/solution/ (2021.07.01 accessed),
14. Sun, P. Z.; Lee, W. N.; Zhang, R. C.; Huang, C. H., Degradation of DEET and caffeine under UV/chlorine and simulated sunlight/chlorine conditions. *Environ. Sci. Technol.* **2016,** *50*, (24), 13265-13273.
15. Guo, K.; Wu, Z.; Shang, C.; Yao, B.; Hou, S.; Yang, X.; Song, W.; Fang, J., Radical chemistry and structural relationships of PPCP degradation by UV/chlorine treatment in simulated drinking water. *Environ. Sci. Technol.* **2017,** *51*, (18), 10431-10439.
16. Benitez, F. J.; Acero, J. L.; Real, F. J.; Roldan, G.; Rodriguez, E., Modeling the photodegradation of emerging contaminants in waters by UV radiation and UV/H2O2 system. *Journal of Environmental Science and Health, Part A* **2013,** *48*, (1), 120-128.
17. Song, W.; Cooper, W. J.; Peake, B. M.; Mezyk, S. P.; Nickelsen, M. G.; O'Shea, K. E., Free-radical-induced oxidative and reductive degradation of N,N´-diethyl-m-toluamide (DEET): Kinetic studies and degradation pathway. *Water Res.* **2009,** *43*, (3), 635-642.
18. APHA, *Standard Methods for The Examination of Water and Wastewater*. 20th ed.; American Water Works Association & Water Environment Federation Washington, D.C., USA 1998.
19. Sander, R. J. A. C.; Physics, Compilation of Henry's law constants (version 4.0) for water as solvent. **2015,** *15*, (8), 4399-4981.
20. Soltermann, F.; Canonica, S.; von Gunten, U., Trichloramine reactions with nitrogenous and carbonaceous compounds: Kinetics, products and chloroform formation. *Water Res.* **2015,** *71*, 318-329.
21. Gligorovski, S.; Strekowski, R.; Barbati, S.; Vione, D., Environmental implications of hydroxyl radicals (• OH). *Chem Rev* **2015,** *115*, (24), 13051-13092.

22. Jing, Y.; Chaplin, B. P., Mechanistic Study of the Validity of Using Hydroxyl Radical Probes To Characterize Electrochemical Advanced Oxidation Processes. *Environ. Sci. Technol.* **2017,** *51*, (4), 2355-2365.
23. Fang, X.; Mark, G.; von Sonntag, C., OH radical formation by ultrasound in aqueous solutions Part I: the chemistry underlying the terephthalate dosimeter. *Ultrason Sonochem* **1996,** *3*, (1), 57-63.
24. Shang, C.; Blatchley, E. R., Differentiation and Quantification of Free Chlorine and Inorganic Chloramines in Aqueous Solution by MIMS. *Environ. Sci. Technol.* **1999,** *33*, (13), 2218-2223.
25. Yagil, G.; Anbar, M., The formation of peroxynitrite by oxidation of chloramine, hydroxylamine and nitrohydroxamate. *Journal of Inorganic and Nuclear Chemistry* **1964,** *26*, (3), 453-460.
26. Beckman, J. S.; Chen, J.; Ischiropoulos, H.; Crow, J. P., [23] Oxidative chemistry of peroxynitrite. In *Methods in Enzymology*, Academic Press: 1994; Vol. 233, pp 229-240.
27. Barker, G.; Fowles, P.; Stringer, B., Pulse radiolytic induced transient electrical conductance in liquid solutions. Part 2.—Radiolysis of aqueous solutions of NO_3^-, NO_2^- and $Fe(CN)_3^{6-}$. *T Faraday Soc* **1970,** *66*, 1509-1519.
28. Pan, X.-M.; Schuchmann, M. N.; Sonntag, C. v., Oxidation of benzene by the OH radical. A product and pulse radiolysis study in oxygenated aqueous solution. *Journal of the Chemical Society, Perkin Transactions 2* **1993**, (3), 289-297.
29. Hand, V. C.; Margerum, D. W., Kinetics and Mechanisms of the Decomposition of Dichloramine in Aqueous-Solution. *Inorg Chem* **1983,** *22*, (10), 1449-1456.
30. Yiin, B. S.; Margerum, D. W., Nonmetal Redox Kinetics - Reactions of Trichloramine with Ammonia and with Dichloramine. *Inorg Chem* **1990,** *29*, (11), 2135-2141.
31. Gordon, S.; Schmidt, K. H.; Hart, E. J., A pulse radiolysis study of aqueous benzene solutions. *The Journal of Physical Chemistry* **1977,** *81*, (2), 104-109.
32. Criquet, J.; Rodriguez, E. M.; Allard, S.; Wellauer, S.; Salhi, E.; Joll, C. A.; von Gunten, U., Reaction of bromine and chlorine with phenolic compounds and natural organic matter extracts – Electrophilic aromatic substitution and oxidation. *Water Res.* **2015,** *85*, 476-486.
33. Bulman, D. M.; Mezyk, S. P.; Remucal, C. K., The impact of pH and irradiation wavelength on the production of reactive oxidants during chlorine photolysis. *Environ. Sci. Technol.* **2019,** *53*, (8), 4450-4459.
34. Chuang, Y. H.; Chen, S.; Chinn, C. J.; Mitch, W. A., Comparing the UV/monochloramine and UV/free chlorine advanced oxidation processes (AOPs) to the UV/hydrogen peroxide AOP under scenarios relevant to potable reuse. *Environ. Sci. Technol.* **2017,** *51*, (23), 13859-13868.
35. Zhang, Z.; Chuang, Y. H.; Huang, N.; Mitch, W. A., Predicting the contribution of chloramines to contaminant decay during ultraviolet/hydrogen peroxide advanced oxidation process treatment for potable reuse. *Environ. Sci. Technol.* **2019,** *53*, (8), 4416-4425.
36. Flyunt, R.; Leitzke, A.; Mark, G.; Mvula, E.; Reisz, E.; Schick, R.; von Sonntag, C., Determination of •OH, $O_2^{\bullet -}$, and hydroperoxide yields in ozone reactions in aqueous solution. *The Journal of Physical Chemistry B* **2003,** *107*, (30), 7242-7253.
37. Asmus, K.; Möckel, H.; Henglein, A. J. T. J. o. P. C., Pulse radiolytic study of the site of hydroxyl radical attack on aliphatic alcohols in aqueous solution. *The Journal of Physical Chemistry* **1973,** *77*, (10), 1218-1221.
38. Hoigne, J.; Bader, H., Rate constants of reactions of ozone with organic and inorganic compounds in water. 1. Non-dissociating organic compounds. *Water Res.* **1983,** *17*, (2), 173-183.
39. Simic, M.; Neta, P.; Hayon, E., Pulse radiolysis study of alcohols in aqueous solution. *The Journal of Physical Chemistry* **1969,** *73*, (11), 3794-3800.
40. Garg, S.; Yuan, Y.; Mortazavi, M.; Waite, T. D., Caveats in the Use of Tertiary Butyl Alcohol as a Probe for Hydroxyl Radical Involvement in Conventional Ozonation and Catalytic Ozonation Processes. *ACS ES&T Engineering* **2022,** *2*, (9), 1665-1676.
